# NME6 is a phosphotransfer-inactive, monomeric NME/NDPK family member and functions in complexes at the interface of mitochondrial inner membrane and matrix

**DOI:** 10.1186/s13578-021-00707-0

**Published:** 2021-11-17

**Authors:** Bastien Proust, Martina Radić, Nikolina Škrobot Vidaček, Cécile Cottet, Stéphane Attia, Frédéric Lamarche, Lucija Ačkar, Vlatka Godinić Mikulčić, Malgorzata Tokarska-Schlattner, Helena Ćetković, Uwe Schlattner, Maja Herak Bosnar

**Affiliations:** 1 Division of Molecular Medicine, Ruđer Bošković Institute, 10000 Zagreb, Croatia; 2 Laboratory of Fundamental and Applied Bioenergetics, Univ. Grenoble Alpes and Inserm U1055, Grenoble, France; 3 The Miroslav Krleža Institute of Lexicography, 10000 Zagreb, Croatia; 4 Division of Molecular Biology, Ruđer Bošković Institute, 10000 Zagreb, Croatia; 5 Univ. Grenoble Alpes and Inserm U1055, Laboratory of Fundamental and Applied Bioenergetics, Grenoble, France, and Institut Universitaire de France (IUF), Paris, France

**Keywords:** NDP kinase, NME, nm23, Mitochondria, RCC1L, WBSCR16

## Abstract

**Background:**

NME6 is a member of the nucleoside diphosphate kinase (NDPK/NME/Nm23) family which has key roles in nucleotide homeostasis, signal transduction, membrane remodeling and metastasis suppression. The well-studied NME1-NME4 proteins are hexameric and catalyze, via a phospho-histidine intermediate, the transfer of the terminal phosphate from (d)NTPs to (d)NDPs (NDP kinase) or proteins (protein histidine kinase). For the NME6, a gene/protein that emerged early in eukaryotic evolution, only scarce and partially inconsistent data are available. Here we aim to clarify and extend our knowledge on the human NME6.

**Results:**

We show that NME6 is mostly expressed as a 186 amino acid protein, but that a second albeit much less abundant isoform exists. The recombinant NME6 remains monomeric, and does not assemble into homo-oligomers or hetero-oligomers with NME1-NME4. Consequently, NME6 is unable to catalyze phosphotransfer: it does not generate the phospho-histidine intermediate, and no NDPK activity can be detected. In cells, we could resolve and extend existing contradictory reports by localizing NME6 within mitochondria, largely associated with the mitochondrial inner membrane and matrix space. Overexpressing NME6 reduces ADP-stimulated mitochondrial respiration and complex III abundance, thus linking NME6 to dysfunctional oxidative phosphorylation. However, it did not alter mitochondrial membrane potential, mass, or network characteristics. Our screen for NME6 protein partners revealed its association with NME4 and OPA1, but a direct interaction was observed only with RCC1L, a protein involved in mitochondrial ribosome assembly and mitochondrial translation, and identified as essential for oxidative phosphorylation.

**Conclusions:**

NME6, RCC1L and mitoribosomes localize together at the inner membrane/matrix space where NME6, in concert with RCC1L, may be involved in regulation of the mitochondrial translation of essential oxidative phosphorylation subunits. Our findings suggest new functions for NME6, independent of the classical phosphotransfer activity associated with NME proteins.

**Supplementary Information:**

The online version contains supplementary material available at 10.1186/s13578-021-00707-0.

## Background

**Fig. 1:**
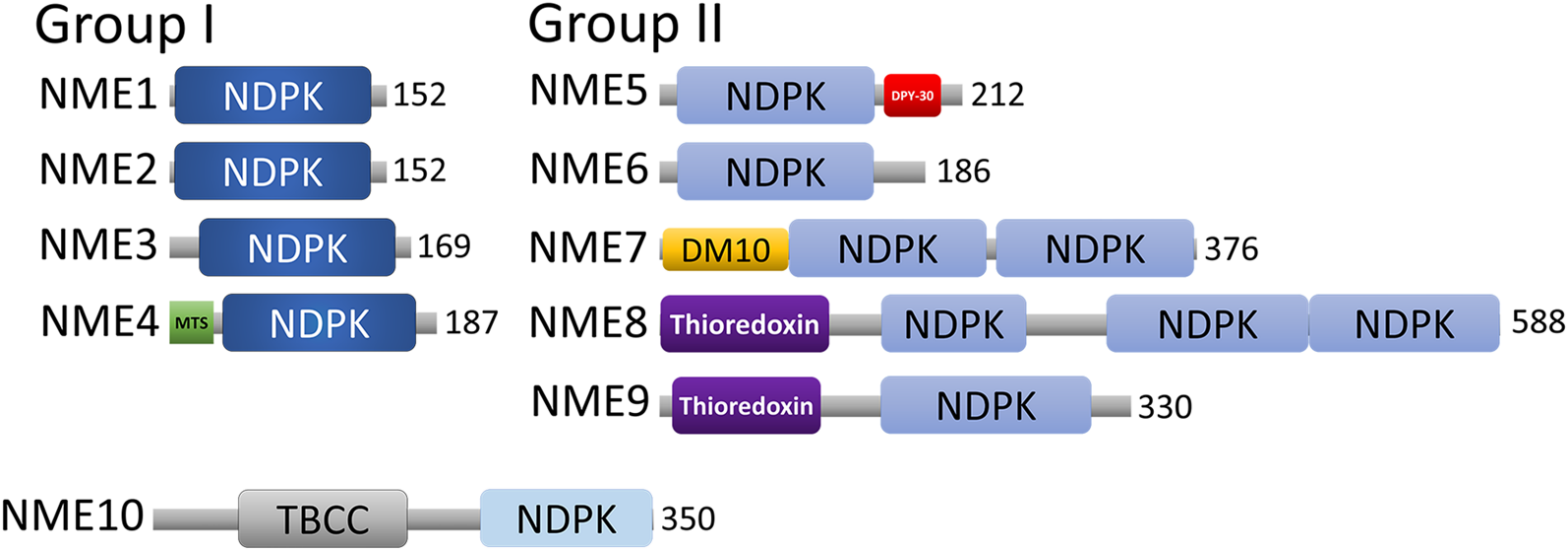
Schematic representation of human NME proteins. The total amino acid length number of the protein is shown at the end of each protein. Protein domains have been indicated with boxes, and each protein has been searched against SMART/Pfam databases. Abbreviations of domain names are retrieved from SMART/Pfam databases and indicated in the figure. Shortened name MTS stands for mitochondrial targeting sequence domain

NME6 is a member of the nucleoside diphosphate kinase (NDPK/NME/Nm23) family of enzymes, responsible for the maintenance of the cellular (d)NTP pool [1]. NDPKs transfer the terminal phosphate group from NTPs (mostly ATP) to all other (d)NDPs through a high-energy phospho-histidine (pHis) intermediate [2]. In human, the family consists of 10 genes divided into two groups on the basis of their gene/protein structure and phylogenetic analysis [3] (Fig. 1). Group I members (NME1-NME4) are highly homologous [4]. They form hexamers and possess the nine conserved amino acid residues essential for protein stability and enzymatic activity [5, 6]. They harbor a single NDPK domain and are catalytically active with similar kinetic parameters [7]. NME1 is able to suppress the metastatic cascade, which made it the first metastasis suppressor gene/protein discovered [8–11]. NME1 and NME2 are highly homologous (88% amino acid identity) [12] and take part in several cellular processes such as apoptosis [13, 14], proliferation [15, 16], differentiation and development [17–19], vesicular trafficking [20], adhesion and migration [21, 22]. While their NDPK activity has been thoroughly documented, both proteins have been assigned other biochemical activities such as protein histidine kinase activity [23], transcriptional regulatory functions [24] or 3’-5’ exonuclease activity [25]. The two remaining Group I members, NME3 and NME4, localize to mitochondria [26–31]. Interestingly, the NME4 is the only human NDPK possessing a typical N-terminal mitochondrial targeting sequence which is cleaved after import [29] (Fig. 1).

In contrast to Group I, research on Group II proteins (NME5-NME9) has been scarce. It seems that the Group II members emerged very early in the eukaryotic evolution [32]. Group II NME members are more divergent among themselves (28–45% amino acid identity) and dissimilar to Group I proteins (25–34% amino acid identity) [4, 33]. Group II proteins display one or more NDPK domains (Fig. 1) and it is unknown whether they also form oligomers [34]. The NME10 does not belong to any of the groups since it seems that its NDPK domain was inserted recently so it has its own evolutionary history [4].

NME6 probably arose by duplication of a NME5-like gene with the emergence of Amoebozoa, such as *Dyctiostelium* [32]. As compared to other NME family members, especially NME1 and NME2, very few studies addressed NME6, although its first description by two separate groups dates back over 20 years. Mehus and coworkers [35] as well as Tsuiki and coworkers [36] both cloned human NME6 cDNAs that showed 34–41% identity with Group I NMEs, located the corresponding gene on chromosome 3p21.3, and detected quite ubiquitous NME6 expression in human tissues. The NME6 sequence of the former study predicted a protein of 186 amino acids (aa) with a pI of 8.5 and a molecular weight of 21.142 kDa. The active site residues were preserved, but there were additional residues inserted into the killer of prune (*Kpn*) loop and at the C-terminal end, leaving open the question whether the NME6 has the potential to oligomerize, and thus display NDPK activity [35]. The study of Tsuiki et al. described a protein with 194 aa. The produced glutathione S transferase (GST) tagged recombinant protein, GST-NME6, showed NDPK activity, but at lower levels than NME1 or NME2, which would make it the only Group II member with enzymatic activity. The study further reported at least partial colocalization of NME6 with mitochondria. The overexpression of NME6 in SAOS2 cells led to growth suppression and formation of multinucleated cells possibly affecting cytokinesis [36]. More recently, large-scale studies detected NME6 in the mitochondrial proteome [28, 37, 38]. Our group was able to isolate the NME6 homolog from the marine sponge *Suberites domuncula*, NME6Sd, which did not possess NDPK activity [39]. In contrast to the human NME6, the sponge NME6Sd contains a putative mitochondrial targeting sequence, but when expressed in human cells did not localize to mitochondria. The sponge NME6Sd lacks two recent introns and has different transcriptional binding sites in the promoter region as compared to human NME6 [4, 39]. Taken together, we still have an incomplete picture of NME6 structure and function, and more information on its molecular and cellular properties is required.

The aim of this study was to identify basic structural and functional features of the barely studied human NME6, using MDA-MB-231T cells, already extensively used as a model system in NME studies [40–43]. We identify two different NME6 isoforms that are expressed in human cells. Both are monomeric, lack NDPK activity and localize to mitochondria, where they associate with the mitochondrial inner membrane (MIM) and the matrix space. Increased NME6 expression levels lead to diminished ADP-stimulated respiration and complex III abundance, while a trend of enhanced ADP-stimulated respiration is observed upon decreased NME6 levels. NME6 associates with different mitochondrial proteins, but stably interacts only with RCC1-like G exchanging factor-like protein (RCC1L) also known as Williams-Beuren syndrome chromosomal region 16 protein (WBSCR16), a putative GDP/GTP exchange factor involved in coordination of mitoribosome assembly and mitochondrial translation [44]. These findings establish NME6 as the third mitochondrial NME, with potentially new and regulatory functions for mitochondrial physiology that are independent of phosphotransfer activity.

## Results

### Endogenous NME6 is predominantly expressed as the 186 aa isoform

**Fig. 2:**
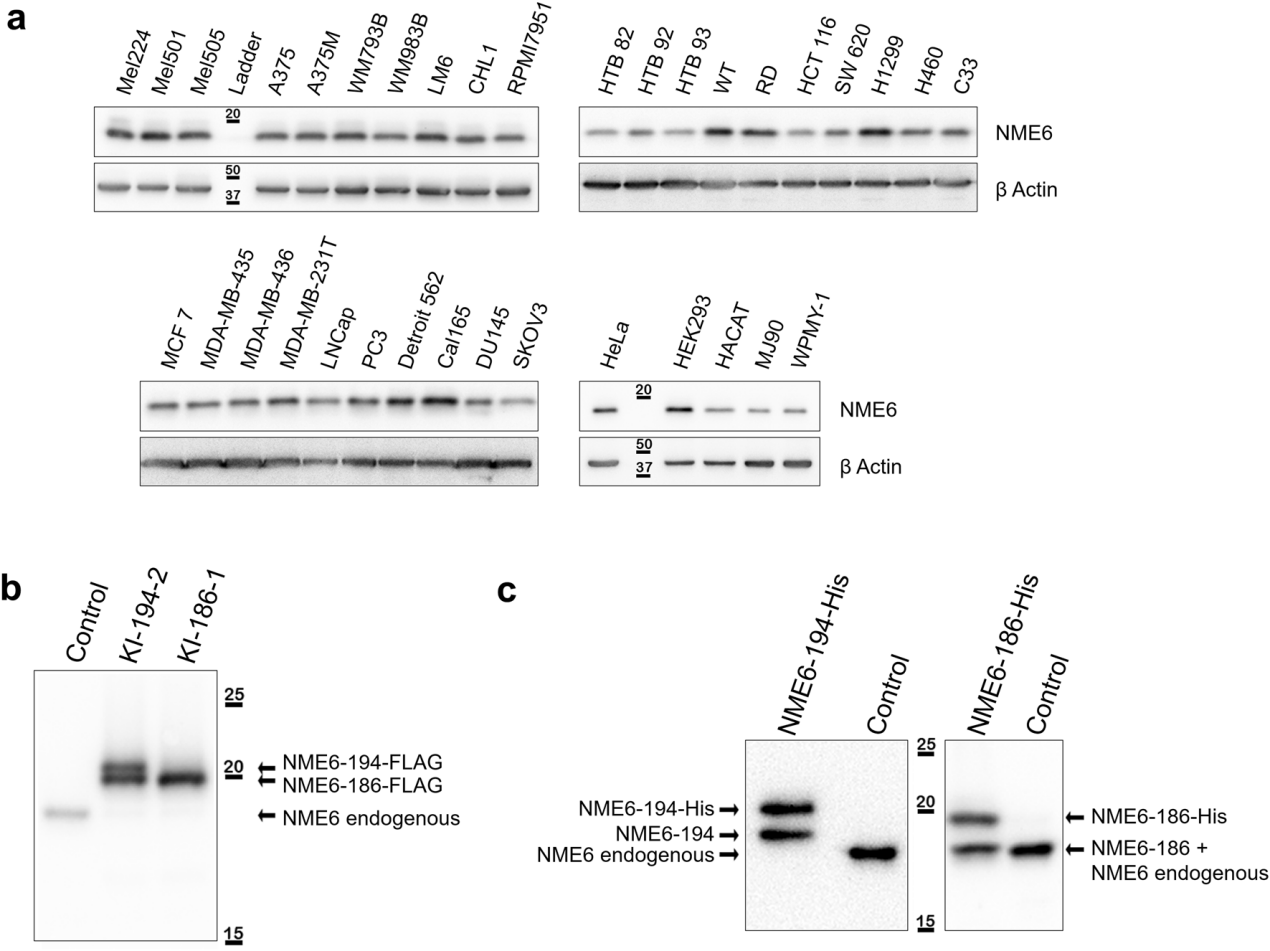
Endogenous NME6 is predominantly expressed as the 186 aa isoform in human cancer cell lines. **a** NME6 expression in a panel of human cancer and non-cancer cell lines. Twenty micrograms of total cell lysate was loaded on SDS-PAGE and analyzed by Western blot using anti-NME6 antibody. **b** Western blot analysis of MDA-MB-231T cell lysates (control) and MDA-MB-231T stable clones overexpressing NME6-194-FLAG (KI-194-2) and NME6-186-FLAG (KI-186-1) proteins, using anti-NME6 antibody. Overexpression of exogenous FLAG-tagged NME6 led to downregulation of endogenous NME6 at the protein level. **c** Western blot analysis of recombinant and endogenous NME6 proteins. Human NME6-194-His and NME6-186-His recombinant isoforms were produced in bacteria and purified. The His-tag was partially removed using thrombin and proteins were analyzed by Western blot along with whole cell lysate from MDA-MB-231T (control), using anti-NME6 antibody. Endogenous NME6 and recombinant NME6-186 display similar migration

To determine the expression profile of NME6 in human, Western blot analysis was performed with the anti-NME6 antibody on a panel of 31 cancerous and 4 non-cancerous cell lines. In all of the tested cell lines of different origin NME6 protein was well detectable (Fig. 2a). The Western blot reveals consistently, for all tested cells, a unique NME6 band, although in human two different NME6 isoforms have been described (194 aa and 186 aa) [35, 36]. Since all the cells examined displayed the same expression pattern we decided to use MDA-MB-231T cells as a major model for our investigation, to describe general characteristics of NME6. These particular cells have been already used as a model system in the NME field. Thus, there is substantial knowledge on NME proteins with these cells [40–42]. To investigate whether the two isoforms can be distinguished on the blot, knock-in (KI) stable clones overexpressing NME6-194-FLAG and NME6-186-FLAG were produced and analyzed by Western blot using the anti-NME6 antibody. The resulting FLAG-tagged proteins are 10–20 times more expressed than endogenous NME6 in control cells (Additional file 2: Fig. S1). The KI of NME6-194-FLAG results in two bands, corresponding to both, the long and the short isoform, showing a visible molecular weight difference on Western blot (respectively above and below 20 kDa). The KI of NME6-186-FLAG gives a single protein band below 20 kDa (Fig. 2b). Endogenous NME6 protein expression decreases strongly in KI clones compared to control cells, indicating a tight regulation of endogenous NME6 expression (Fig. 2b). To reveal which of the two isoforms predominates in the analyzed cells, we performed an additional Western blot comparing the molecular weight of the endogenous NME6 with both recombinant protein isoforms produced in bacteria (NME6-194-His and NME6-186-His), after partial thrombin-cleavage of the His-tag residue. Results show that endogenous NME6 migrates at the same molecular weight as the NME6-186 isoform while a clear shift is observed between the endogenous protein and NME6-194 (Fig. 2c), strongly indicating the shorter isoform to be predominantly expressed in human cells. Since our immunoblotting experiments revealed only one endogenous NME6 band in different cell lines, we used mass spectrometry (MS) for independent confirmation (Additional file 3: Fig. S2). MS detected both, endogenous NME6-194 and NME6-186 in HeLa and MDA-MB-231T cells. Thus, human cells can express both isoforms, but the longer protein isoform is much less abundant, escaping immunoblot detection.

### NME6 lacks NDP kinase activity, and does not form homo-oligomers

**Fig. 3:**
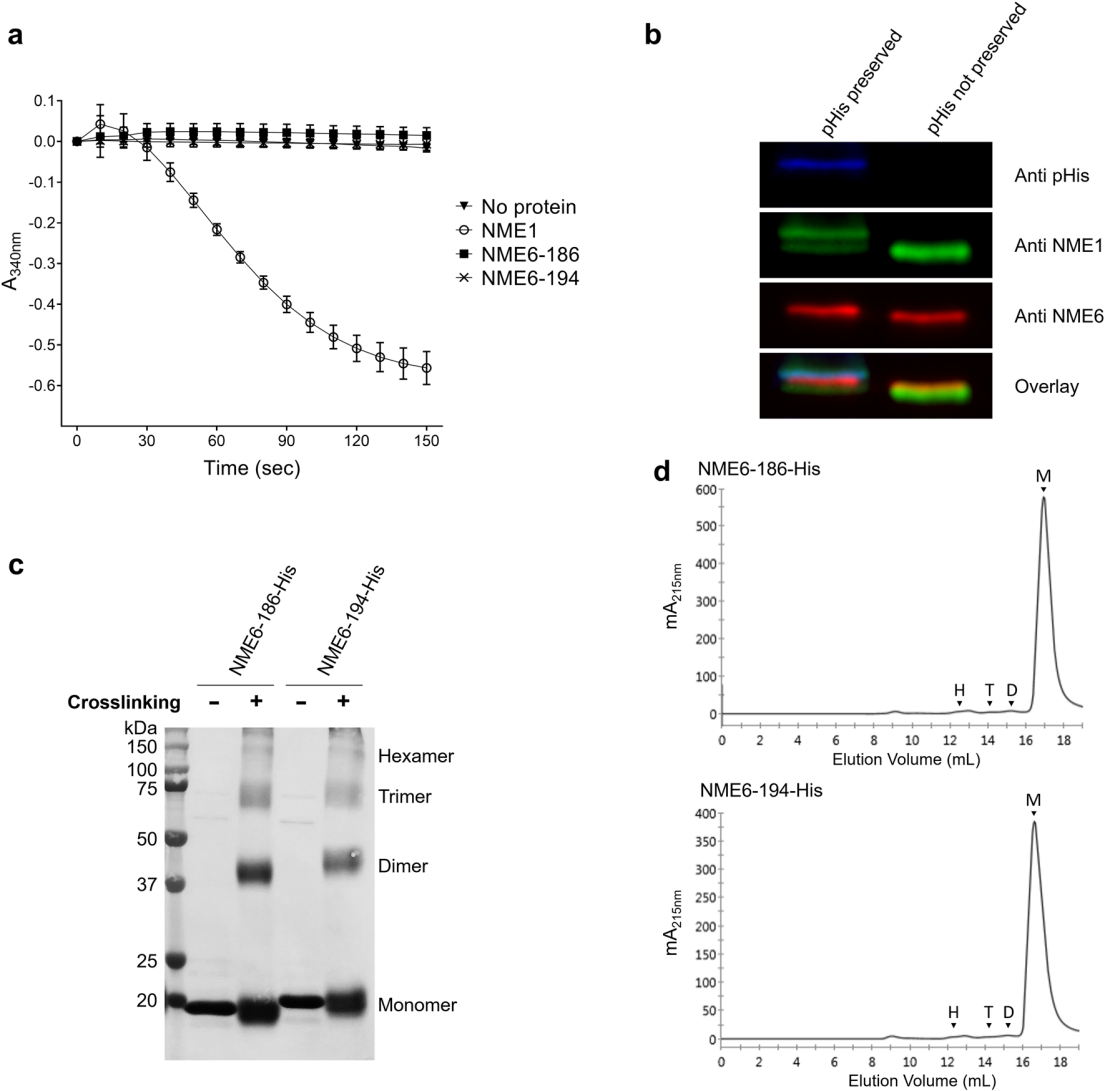
NME6 proteins lack NDP kinase activity and exhibit preferentially the monomeric state. **a** Recombinant NME6 proteins lack NDP kinase activity. Purified NME1-His (positive control), NME6-186-His and NME6-194-His recombinant proteins were subjected to an enzymatic activity assay where the decrease of NADH absorbance at 340 nm over time is directly proportional to NDP kinase activity (n = 6). **b** Endogenous NME6 lacks histidine phosphorylation. MDA-MB-231T cell lysate was immunoblotted with and without preserving histidine phosphorylation, using anti-NME1, a mix of anti-phospho-histidine 1-pHis and 3-pHis and anti-NME6 antibodies. Note: A single overlapping signal is observed only in preserving conditions between NME1 and pHis (turquoise), corresponding to pHis-NME1 (positive control). **c** NME6 recombinant proteins remain mostly monomeric under crosslinking conditions. Glutaraldehyde crosslinking with pure recombinant NME6-186-His or NME6-194-His proteins was analyzed by Western blotting and protein staining with naphthol blue. **d** NME6 recombinant proteins are mostly monomeric. Size exclusion chromatography was performed by loading purified recombinant proteins NME6-186-His and NME6-194-His onto a Superdex 200 Increase 10/300 GL column, calibrated for molecular mass by a series of standard proteins. The protein elution profile was recorded by absorbance at 215 nm. The elution volume of monomers (M), dimers (D), trimers (T) and hexamers (H) expected from the calibration is indicated by arrows

A key property of Group I NME proteins is their NDPK activity, dependent on the phosphorylation of a specific histidine residue in the catalytic site. Further, it seems that the formation of homo- or heterohexamers is a prerequisite for NDPK activity in eukaryotes [45–47]. Recombinant NME6-186-His and NME6-194-His isoforms were tested for NDPK activity by the standard pyruvate kinase-lactate dehydrogenase coupled assay with dTDP as a phosphate acceptor [2] (Fig. 3a). Human recombinant NME1-His (positive control) shows an NDPK activity of 155 U/mg, while a reaction mix deprived of protein was used as a negative control. Neither of the two recombinant NME6 isoforms showed enzymatic activity (Fig. 3a). We then attempted to detect the phosphorylated histidine in the endogenous NME6, a prerequisite for NDPK activity. The MDA-MB-231T cell lysate was subjected to Western blot under pHis preserving and non-preserving conditions, using either an antibody mix against 1-pHis and 3-pHis, or anti-NME1, or anti-NME6 (Fig. 3b). NME1 detection revealed two bands under pHis preserving conditions and a single band under non-preserving conditions. The higher NME1 migrating band entirely superposes with the single pHis band in preserving conditions (overlay, turquoise color) and thus corresponds to phosphorylated NME1 (pHis-NME1). For NME6, only a single band is detected under both conditions. Under pHis preserving conditions, the NME6 band does not superimpose with the pHis band. These results show that, contrary to NME1, endogenous NME6 lacks phosphorylation on histidine residues, including the active site histidine, crucial for NDPK activity. Further, we analyzed the oligomerization status of recombinant NME6 isoforms (Fig. 3c, d). Recombinant NME6-186-His and NME6-194-His were subjected to crosslinking using glutaraldehyde (Fig. 3c), forcing the formation of covalent bonds [48]. Results show that the largely predominant structure of both NME6 isoforms is monomeric. A probably dimeric structure can also be detected, although to a much lesser extent. Structures of higher molecular mass, including hexamers essential for NDPK activity, are very minor (Fig. 3c). These findings were confirmed by size exclusion chromatography performed on purified recombinant isoforms. The chromatograms reveal the almost exclusive presence of the monomeric subpopulation for both NME6-194-His and NME6-186-His isoforms (95.8% and 96%, respectively) (Fig. 3d). There is no noticeable difference in the oligomerization properties between the two isoforms.

### Endogenous NME6 does not physically interact with NME Group I proteins

**Fig. 4:**
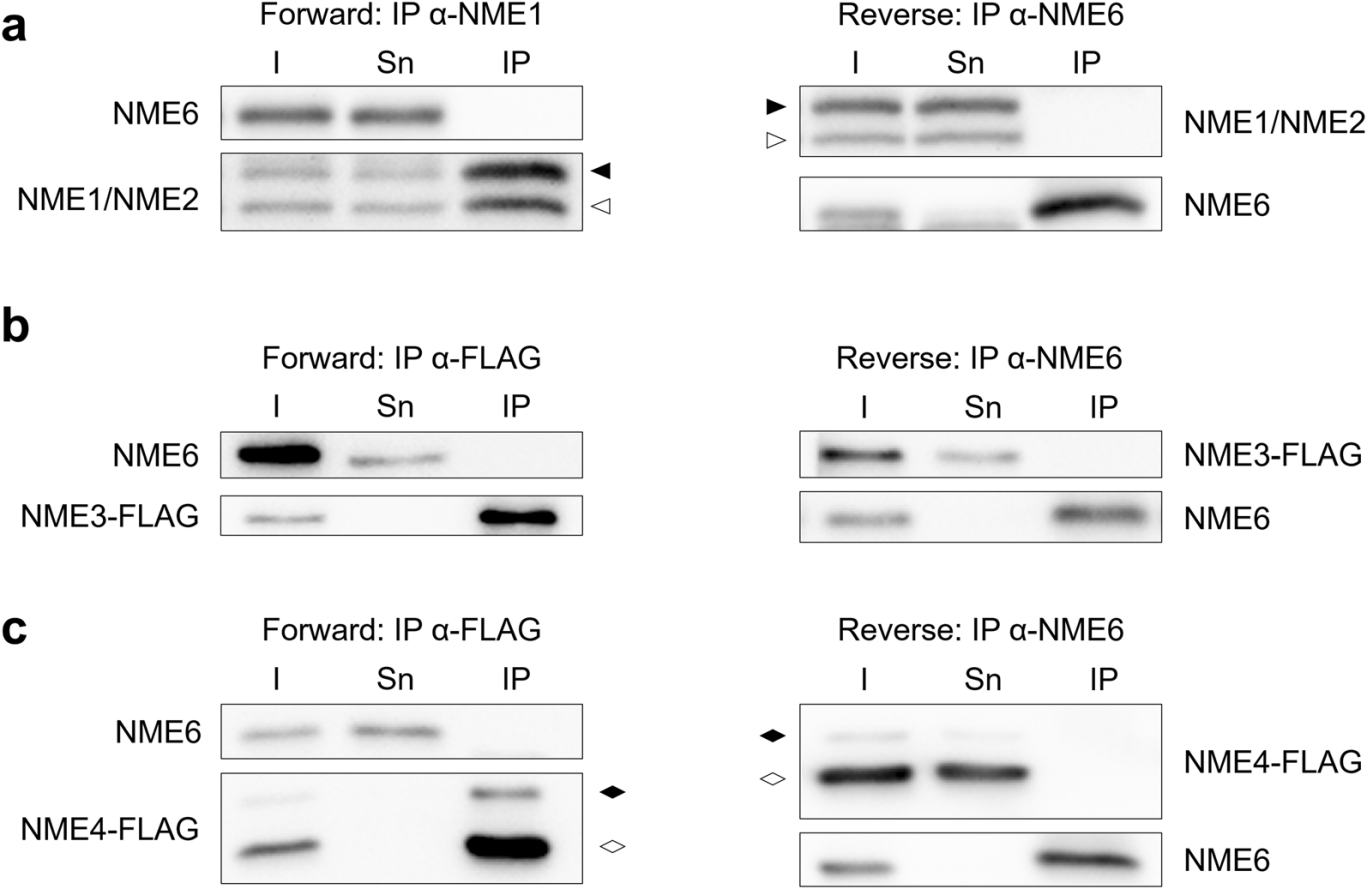
Endogenous NME6 does not interact with Group I NME proteins. Co-immunoprecipitations were performed in a forward and reverse manner between Group I NME proteins and endogenous NME6 protein in MDA-MB-231T cells. Input proteins (I), supernatant depleted of immunoprecipitated proteins (Sn) and eluted proteins (IP) were analyzed by Western blot. **a** Endogenous NME proteins were immunoprecipitated using anti-NME1 or anti-NME6 coupled with Dynabeads™ protein G. Western blots were done using anti-NME1/2 antibody against NME1 () and NME2 (), and anti-NME6. Proteins from cells transiently transfected to express **b** NME3-FLAG or **c** NME4-FLAG were pulled-down using FLAG-agarose or anti-NME6 coupled with Dynabeads™ protein G. Western blots were revealed using anti-FLAG and anti-NME6. NME4-FLAG displays two bands corresponding to NME4-full-lenght () and NME4-truncated after mitochondrial import (). NME6 shows no interaction with Group I NME proteins

NME proteins, mainly NME1 and NME2, have been shown to form both, homo and heterohexamers [12, 49, 50]. Since we have shown that NME6 does not form homo-oligomers, we investigated the possible hetero-oligomerization of NME6 with Group I NME members. For this purpose, immunoprecipitations (IP) were performed in a forward and reverse manner using MDA-MB-231T cell lysates (Fig. 4). NME1 was immunoprecipitated using a highly specific antibody (OP48, Calbiochem) and revealed with an antibody that recognizes both, NME1 and NME2. (Fig. 4a). Western blot analysis reveals that NME1 forms complexes with NME2, as expected, but not with NME6 (Fig. 4a, forward). The reverse experiment confirms the lack of physical interaction between endogenous NME6 and endogenous NME1 and/or NME2 (Fig. 4a, reverse). Endogenous NME3 and NME4 display a low expression level in MDA-MB-231T cells. Therefore, the exogenous NME3-FLAG (Fig. 4b) and NME4-FLAG (Fig. 4c) were expressed in MDA-MB-231T cells, and pulled-down using FLAG-agarose. Expression of NME4-FLAG generates two proteins, a full-length protein and a truncated form due to cleavage of the mitochondrial targeting sequence following mitochondrial import [29]. In the pull-down with FLAG-tagged NME3 or NME4, no physical interaction with NME6 protein is detectable. Likewise, the reverse NME6 IP confirmed the lack of physical interaction with either NME3-FLAG or NME4-FLAG. (Fig. 4b, c, respectively). In conclusion, our findings demonstrate that NME6 does not interact with any of the Group I NME protein members in MDA-MB-231T cells.

### NME6 localizes inside mitochondria, associated with inner membrane and matrix

**Fig. 5:**
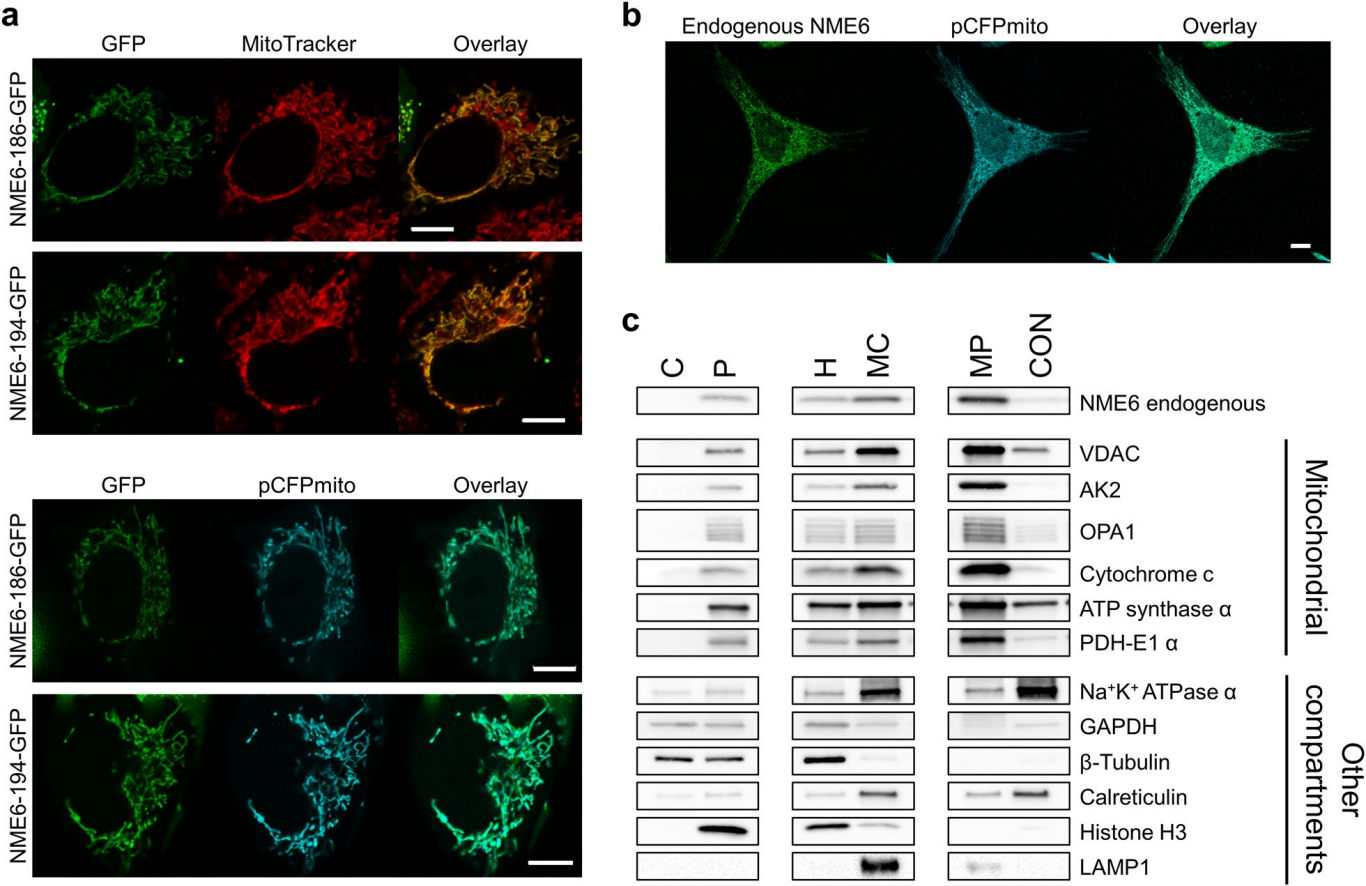
NME6 localizes in mitochondria. **a** Exogenous NME6-GFP colocalizes with mitochondria in living cells. MDA-MB-231T cells transfected with pEGFPN1-NME6-186-GFP and pNME6-194-GFP were stained with MitoTracker™ (red) or cotransfected with pCFPmito to label mitochondria. Images were acquired 48 h post-transfection by confocal microscopy (scale bar: 10 µm). **b** Endogenous NME6 colocalize with mitochondria in fixed cells. HeLa cells transfected with pCFPmito to label mitochondria (cyan) were fixed and exposed to anti-NME6 antibody (green), before acquisition by confocal microscopy (scale bar: 10 µm). **c** Endogenous NME6 is enriched in Percoll-purified mitochondria. MDA-MB-231T cell fractionation was performed by differential centrifugation of cell homogenate (H) followed by a purification of crude mitochondria (MC) on Percoll gradient (C: cytosol, P: nucleus-rich pellet, MP: Percoll-purified mitochondria, CON: major contaminating band recovered after Percoll gradient). Fractions were evenly loaded on SDS-PAGE and analyzed by Western blot using antibodies against mitochondrial protein markers, markers for other compartments and NME6

At the cellular level, we were first interested to identify the precise localization of NME6 in MDA-MB-231T and HeLa cells. Confocal microscopy of living (Fig. 5a) and fixed cells (Fig. 5b) indicated mitochondrial localization. Cells transfected with pCFPmito to express a mitochondrial cyan fluorescent protein (CFP), then fixed and immunostained for NME6, showed overlapping fluorescence of mitochondria and endogenous NME6 (Fig. 5b). Further, MDA-MB-231T cells transiently transfected to express NME6-194-GFP or NME6-186-GFP were labeled for mitochondria by either co-transfection with pCFPmito (Fig. 5a, bottom panels) or live-cell labeling with MitoTracker™ Deep Red FM (Fig. 5a, top panels). Live cells analyzed 48 h post-transfection again showed overlay of mitochondria and NME6 fluorescence. Time-lapse microscopy demonstrated that NME6 retains the mitochondrial localization in HeLa, MDA-MB-231T and RKO cell lines during 24 h (Additional files 4, 5 and 6: Movies S1, S2 and S3, respectively). As a second, independent approach we performed different types of cell fractionation. An initial crude detergent-based approach yielding fractions enriched in cytosol, mitochondria and nuclei detected NME6 only in mitochondria, together with the mitochondrial markers ATP synthase α and pyruvate dehydrogenase E1 component subunit alpha (PDH-E1 α) (Additional file 7: Fig. S3). These results were confirmed by a second fractionation based on differential centrifugation, followed by mitochondrial purification on a Percoll gradient. Again, NME6 was enriched in Percoll-pure mitochondria (MP), together with a panel of mitochondrial markers (Fig. 5c).

**Fig. 6:**
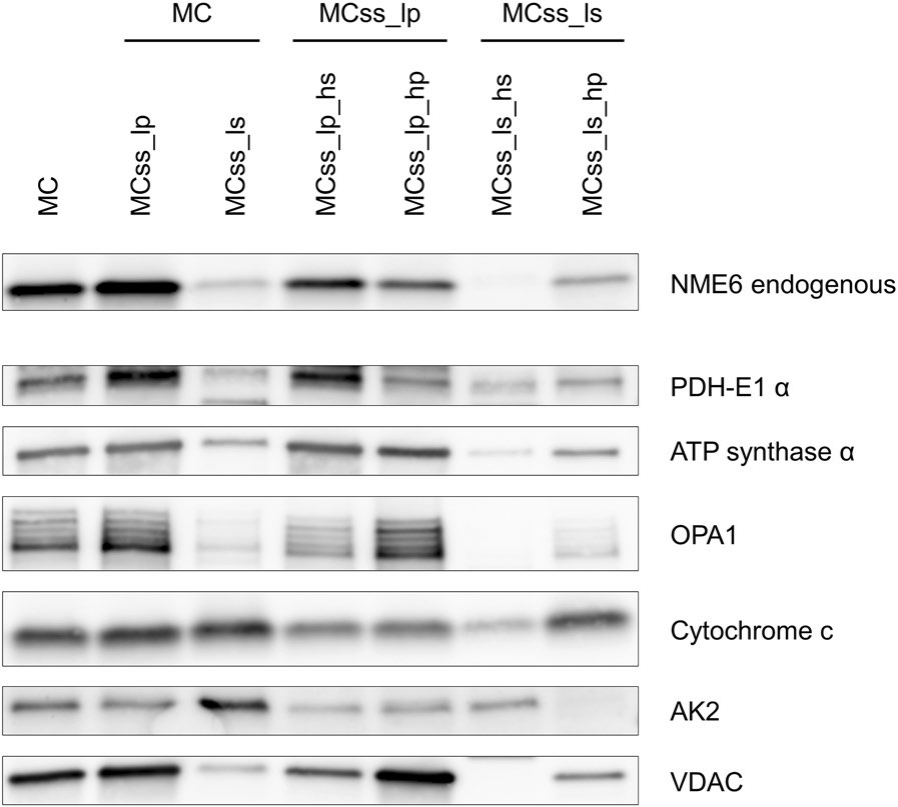
Sub-mitochondrial NME6 distribution is typical for proteins associated with the inner membrane and matrix space. Crude mitochondria (MC) from MDA-MB-231T cells were subfractionated by a two-step protocol. First, swelling-shrinking followed by mild sonication and low speed centrifugation separates mitochondrial inner compartment (pellet MCss_lp, mainly matrix and MIM) and outer compartment (supernatant MCss_ls, mainly cristae/IMS and MOM). Second, high speed centrifugations separate from each of these two fractions the soluble components (supernatants _hs) and the membrane-bound components (pellets _hp; for details see “Materials and methods”). Fractions were evenly loaded on SDS-PAGE and analyzed by immunoblotting for NME6 and different protein markers for mitochondrial subcompartments. These include: pyruvate dehydrogenase E1 component subunit alpha (PDH-E1 α; matrix), ATP synthase subunit α (a part of the peripheral F_1_ subcomplex of the MIM ATP synthase, facing matrix space), optic atrophy 1 (OPA1; transmembrane MIM, facing IMS), cytochrome *c* (peripherally bound to MIM and soluble in IMS), adenylate kinase 2 (AK2; soluble in IMS) and voltage-dependent anion-selective channel (VDAC; MOM and MIM-MOM contact sites)

Finally, to obtain insight into submitochondrial localization of NME6, we performed subfractionation of washed, crude mitochondria (MC) from MDA-MB-231T cells. First, we used a swelling-shrinking procedure followed by mild sonication and low-speed centrifugation, resulting in low-speed pellet and supernatant, corresponding to inner and outer mitochondrial compartments, respectively (Fig. 6, left part). The low-speed pellet was highly enriched in well-established MIM and matrix marker proteins (OPA1, ATP synthase α, PDH-E1 α) and the low-speed supernatant was clearly enriched in the soluble intermembrane space (IMS) marker adenylate kinase 2 (AK2), and also contained mitochondrial outer membrane (MOM) marker voltage-dependent anion-selective channel protein (VDAC), although a majority was retained in the pellet due to MIM-MOM contact sites. Cytochrome *c*, which is bound to MIM but also soluble in the IMS, was partitioned between pellet and supernatant. Importantly, NME6 was detected mainly in the pellet, clearly associating the protein with the inner mitochondrial compartment (MIM and matrix). Second, the low-speed supernatant and pellet were further subfractionated by high-speed centrifugation, to differentiate between soluble (supernatants) and stably membrane-bound components (pellets) of the inner and outer mitochondrial compartments (Fig. 6, right part). Here, NME6 distribution among fractions resembles the one of ATP-synthase-α, a subunit of the MIM complex facing the matrix space, and PDH-E1-α, a matrix protein partially bound to MIM. These data suggest that the large majority of NME6 is a matrix protein, partially bound to MIM (i.e. peripherally bound, facing the matrix space).

### NME6 overexpression negatively affects ADP-stimulated respiration

**Fig. 7:**
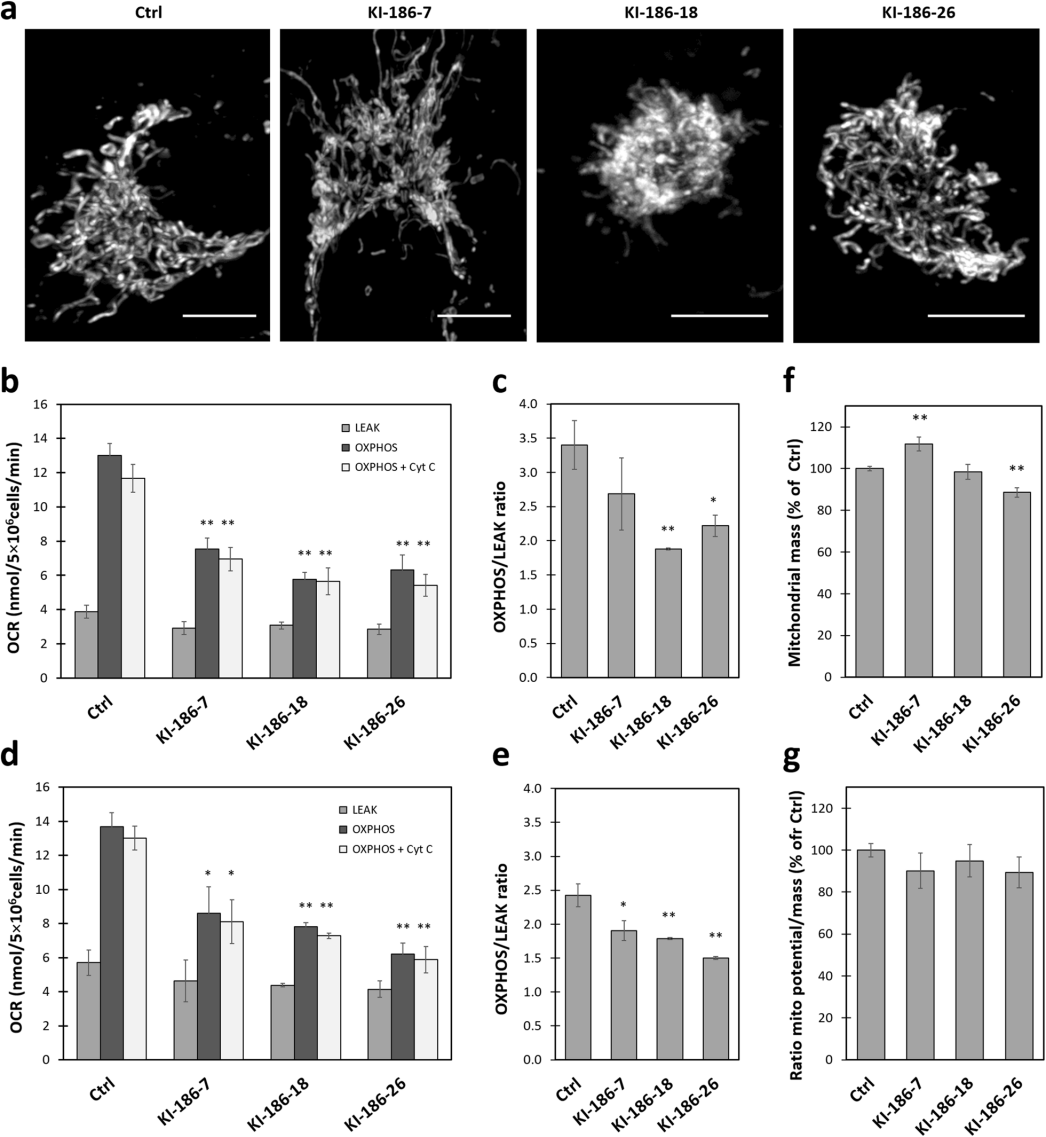
NME6 overexpression affects cellular distribution and ADP-stimulated respiration of mitochondria, but not their membrane potential. **a** Confocal images of MDA-MB-231T cells stained with Mitotracker Green, either wild-type (Ctrl) or clones stably overexpressing NME6-186-FLAG about 10-times (KI-186-7) or about 20-times (KI-186-18 and -26) as compared to endogenous NME6 in Ctrl (scale bar: 10 µm). 3D animations of all shown cells are available as Additional files 8, 9, 10, 11: Movie S4, S5, S6, S7. Note the altered distribution of mitochondria in particular in KI-186-18 and -26 cells, forming dense clusters around the nuclei. **b–e** Oxygraphy analysis of respiration in the cells shown in (**a**) that were digitonin-permeabilized and supplied with substrate (LEAK), stimulated with ADP (OXPHOS), and tested for mitochondrial membrane integrity (addition of cytochrome *c*). **b** Cellular oxygen consumption with glutamate/malate. **c** OXPHOS/LEAK ratios for **b**. **d** Cellular oxygen consumption with succinate. **e** OXPHOS/LEAK ratios for **d**. **f** Mitochondrial mass determined by Mitotracker Green staining. **g** Mitochondrial membrane potential determined by TMRM and corrected for mitochondrial mass (for details see “Material and methods”). All data are given as mean ± SEM (n = 3). For comparison between Ctrl and NME6 overexpressing clones, significance is given as **p < 0.01; *p < 0.05 (Student’s test)

Having localized NME6 at least partially in the functionally important MIM, we verified some basic structural and functional parameters of mitochondria under conditions of graded NME6 overexpression. We used clones stably overexpressing NME6-FLAG at about tenfold (KI-186-7) to 20-fold (KI-186-18, and KI-186-26) higher levels than endogenous NME6 in wild-type (Ctrl) cells (Additional file 2: Fig. S1). Visual inspection of confocal images revealed that mitochondria in cells with high NME6 expression were more concentrated in perinuclear regions (Fig. 7a) (Additional file 12: Fig. S4a). However, the mitochondrial shape and network parameters as shown for the elongation factor remained unchanged (Additional file 12: Fig. S4c). NME6 overexpression seems to relocalize mitochondria close to the nucleus, without affecting the mitochondrial fusion/fission equilibrium.

**Fig. 8:**
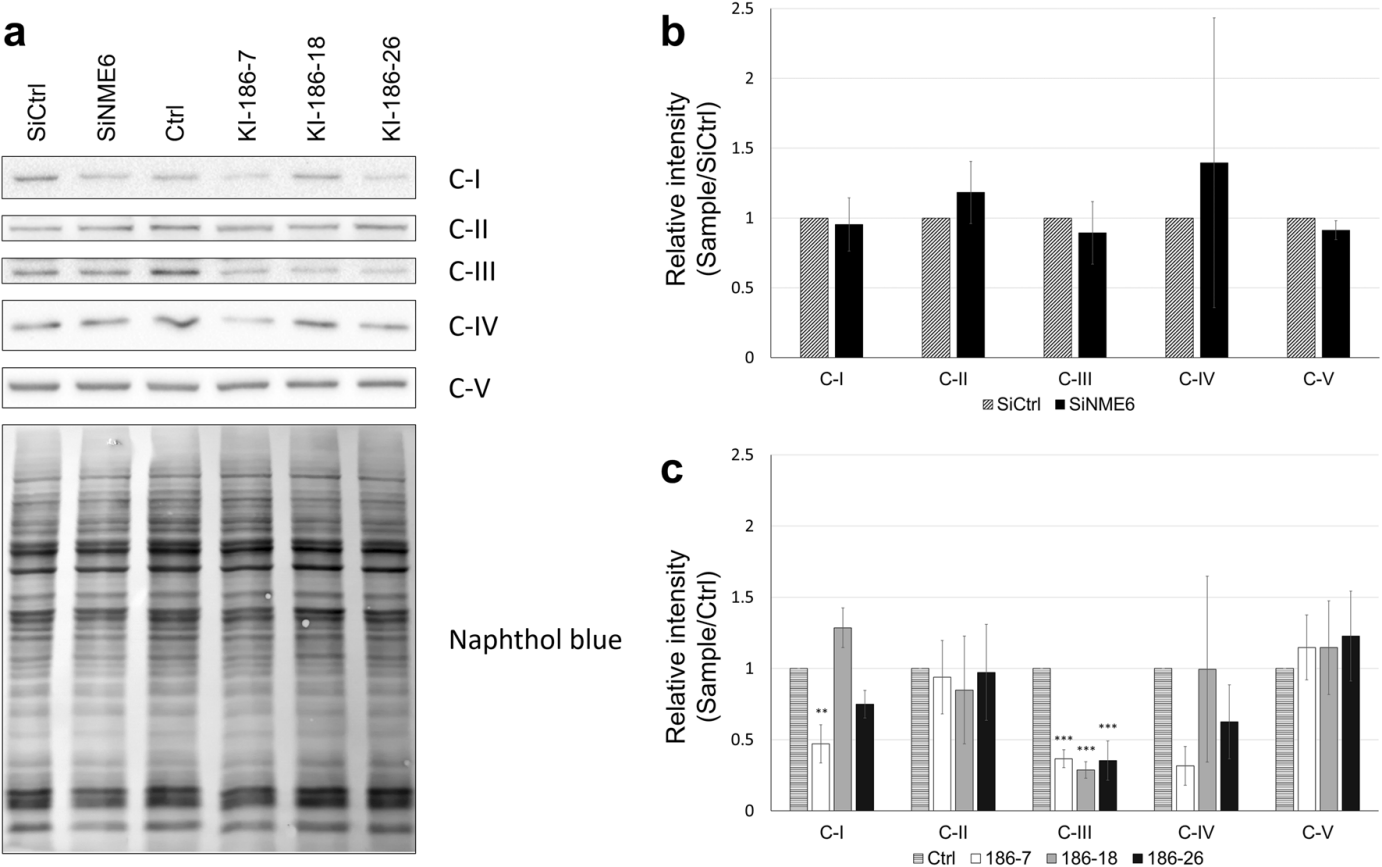
Stable overexpression of NME6 does not affect mitochondrial complex II, but downregulates complex III protein abundance. **a** Ten micrograms of cell lysate from MDA-MB-231T (Ctrl), silenced with scramble control (siCtrl), silenced for NME6 (siNME6) and stable clones overexpressing NME6-186-FLAG (KI-186-7, KI-186-18, KI-186-26) were analyzed by Western blot, using an antibody cocktail against OXPHOS complexes C-I to C-V (ab110412). Band intensity values were normalized to naphthol blue staining, and displayed as a ratio relative to **b** siCtrl or **c** Ctrl. The experiment was realized in triplicate (n = 3). Statistical analysis was performed using **b** Student’s test or **c** one-way ANOVA. All data are given as mean ± SD. Significances are given as ** p < 0.01; *** p < 0.001

We then measured respiration in digitonin-permeabilized cells using the complex I-linked substrates glutamate/malate (Fig. 7b, c) or the complex II-linked substrate succinate (Fig. 7d, e). In both cases, respiration with substrate (LEAK) only was similar. However, after addition of ADP (OXPHOS), significant differences occurred (Fig. 7b, d). All NME6-FLAG overexpressing clones showed reduced ADP-stimulated respiration, leading to lower OXPHOS/LEAK ratios (Fig. 7c, e). The altered ADP-stimulated respiration had no effect on the mitochondrial membrane potential and was not due to lower mitochondrial mass, which both were similar between control cells and overexpressing clones (Fig. 7f, g). However, expression analysis of OXPHOS complexes by Western blot (Fig. 8) revealed downregulation of complex III (CIII) in all KI clones, and a tendency to downregulation of complexes I and IV (CI and CIV) in some KI clones. Abundance of complexes II and V is unchanged with NME6 overexpression.

We then analyzed the same parameters for MDA-MB-231T cells, where NME6 was silenced to about 85% (Additional file 13 Fig. S5a, b). ADP-stimulated respiration (OXPHOS) increases slightly when compared to control for both complex I- and complex II-linked substrates (Additional file 13: Fig. S5d, f), but this did not lead to statistically significant differences in OXPHOS/LEAK ratios (Additional file 13: Fig. S5e, g). No statistical differences between NME6 silenced cells and control cells are observed for mitochondrial mass, membrane potential (Additional file 13: Fig. S5h, i), network parameters (Additional file 12: Fig. S4b, d) or OXPHOS complexes abundance (Fig. 8b).

### NME6 association with mitochondrial proteins

**Fig. 9:**
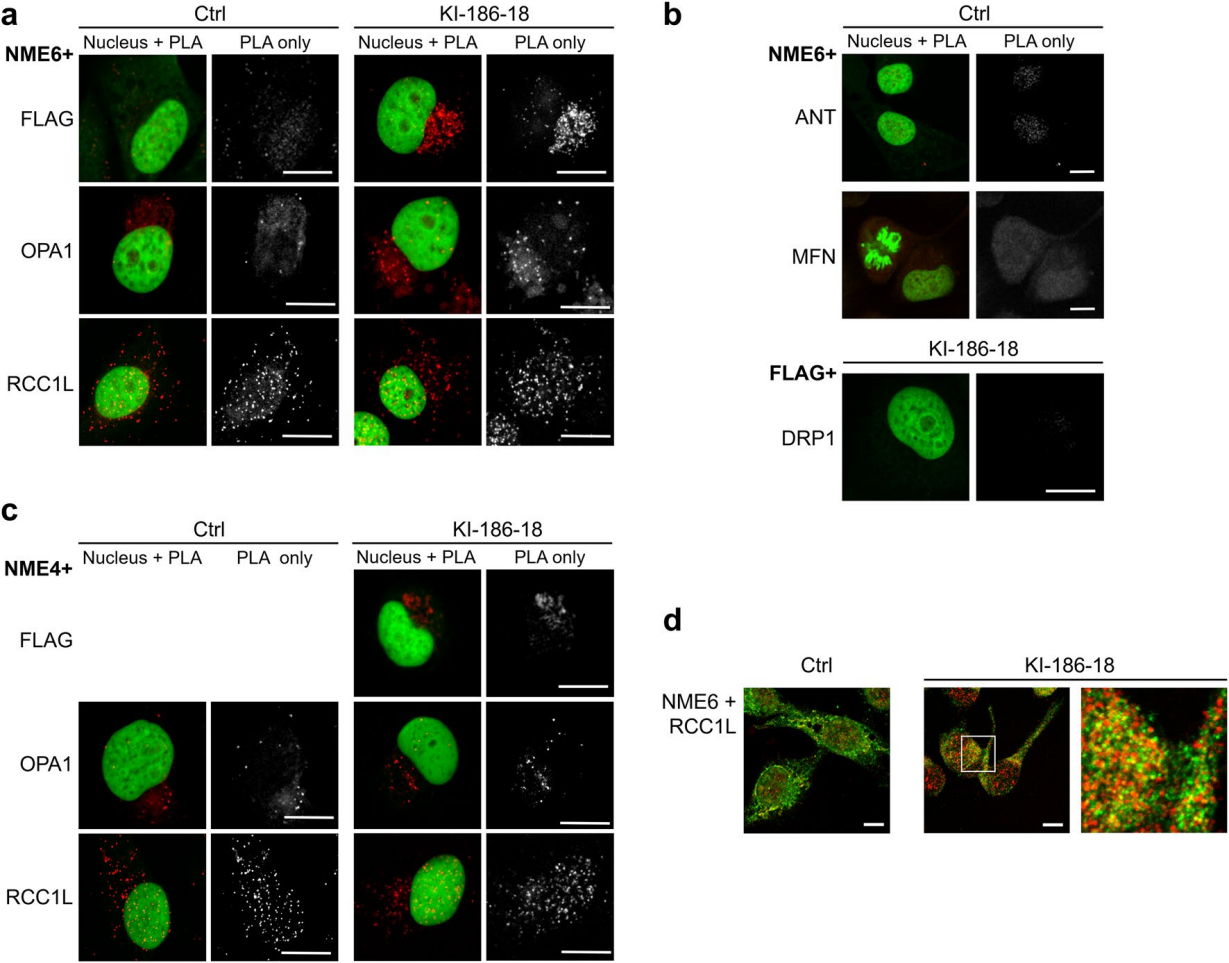
Proximity ligation assays reveal association of NME proteins with OPA1 and RCC1L. **a–c** Proximity ligation assays (PLA) using MDA-MB-231T cells (Ctrl) or clones stably overexpressing NME6-186-FLAG (KI-186-18). Pictures show merged nuclear (green) and PLA stain (red), and PLA stain only (grey). PLA directed against **a** NME6/FLAG-NME6, NME6/OPA1 and NME6/RCC1L, **b** NME6/ANT, NME6/MFN and FLAG-NME6/DRP1, **c** NME4/FLAG-NME6, NME4/OPA1 and NME4/RCC1L. **d** Dual immunofluorescence stain of NME6 (green) and RCC1L (red) revealing colocalization in both Ctrl and KI-186-18 cells. PLA for NME6/FLAG-NME6 identifies two epitopes on the same protein as in KI-186-18 cells (positive control), but fails when FLAG-NME6 is lacking as in Ctrl cells (negative control). The NME4/FLAG PLA detects NME4/NME6 association, but is only applicable to KI-186-18 cells. (All scalebars: 10 µm)

Finally, to gain more insight into putative NME6 functions, we screened for potential NME6 association partners in mitochondria of MDA-MB-231T cells. We applied antibody-based proximity ligation assay (PLA) to analyze the consistent and close proximity between NME6 and a panel of mitochondrial candidate proteins (Fig. 9a, b; for PLA controls see Additional file 14: Fig. S6). In addition to wild-type (Ctrl) cells, we used MDA-MB-231T clones stably overexpressing FLAG-tagged NME6 (KI-186-18) to increase interaction probability and to gain an additional epitope for PLA. The FLAG-NME6 expressing KI-186-18 cells tested with anti-FLAG and anti-NME6 antibodies as a positive control gave a strong PLA signal (Fig. 9a). In contrast, one of the most abundant proteins of the MIM, ADP/ATP translocase (ANT), did not yield PLA signals with NME6 (Fig. 9b). These data support a good specificity of the PLA assay. We next tested three dynamin-related GTPases involved in mitochondrial membrane dynamics, namely mitofusin1/2 (MFN) and dynamin-related protein 1 (DRP1), both located at the outer face of MOM, as well as OPA1 at the outer face of MIM. Earlier studies revealed interaction of DRP1 with NME3 [26] and of OPA1 with NME4 [31], both important for efficient fueling of these GTPases with GTP. In MDA-MB-231T cells, MFN and DRP1 did not show association with NME6, consistent with their MOM localization (Fig. 9b). In contrast, MIM-protein OPA1 did show positive PLA signals with NME6 (Fig. 9a). The same was observed for NME4/FLAG-NME6 in KI-186-18 cells, likely due to the presence of OPA1/NME4 complexes (Fig. 9c). For further candidate proteins, we analyzed data from large-scale studies that mapped physical or functional protein–protein interactions in mitochondria [37, 51–54]. The only putative NME6 interactor that was consistently detected is RCC1L, also known as WBSCR16.

**Fig. 10:**
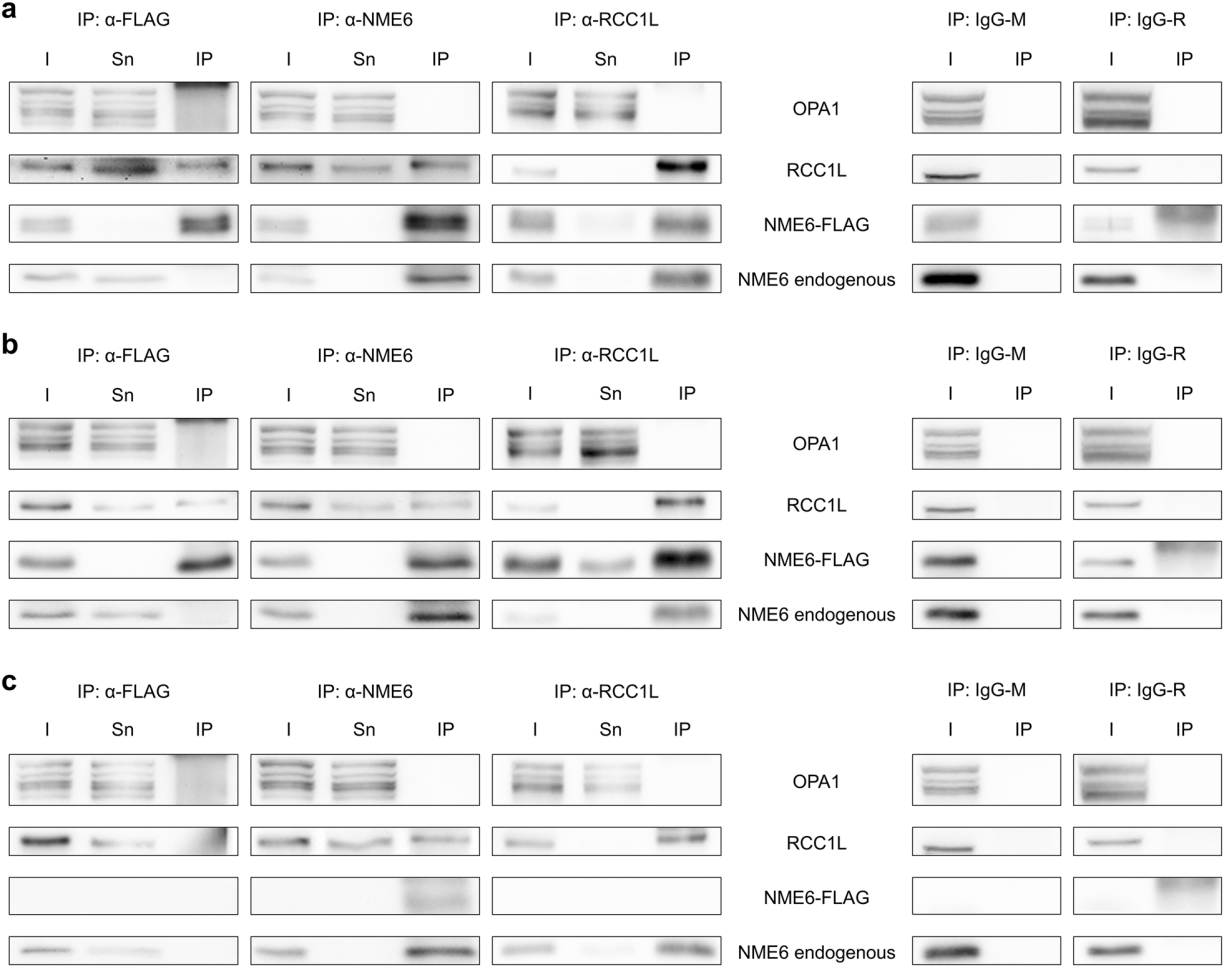
Co-immunoprecipitation of RCC1L and NME6 reveal physical interaction. MDA-MB-231T cells were either transiently transfected to express **a** NME6-194-FLAG or **b** NME6-186-FLAG, or **c** non-transfected control cells were used. Cell lysates were subjected to pull-down with FLAG-agarose, or anti-NME6 and anti-RCC1L antibodies coupled to protein G-Dynabeads™. Immunoprecipitation with irrelevant immunoglobulins from mouse (IgG-M) or from rabbit (IgG-R) were used as a negative control. Input proteins (I), supernatant depleted of pulled-down proteins (Sn) and eluted proteins (IP) were analyzed by immunoblot with anti-OPA1, anti-RCC1L and anti-NME6 antibodies. Both endogenous and FLAG-NME6 co-immunoprecipitate with RCC1L, but not OPA1. Anti-FLAG does not immunoprecipitate endogenous NME6, confirming that NME6 does not assemble into higher homo-oligomers

This protein is a putative GDP/GTP exchange factor bound to MIM [44]. According to recent reports, RCC1L can interact at the outer face of MIM with OPA1 to regulate MIM fusion [55]. It has also been found at the inner (matrix) face associated with mitoribosome subunits, with a role in mitoribosome assembly [44] and/or the intra-mitochondrial translation machinery [51]. Indeed, the PLA assay was positive for NME6/RCC1L in both Ctrl and KI-186-18 cells; interestingly, also NME4 was PLA-positive for RCC1L (Fig. 9a, c). We finally tested whether NME6 occurs in stable complexes with OPA1 and/or RCC1L by using IP from extracts of MDA-MB-231T cells transiently expressing NME6-194-FLAG (Fig. 10a), NME6-186-FLAG (Fig. 10b) or none of both (Fig. 10c). IPs pulled down either FLAG-tagged NME6 (left panels), total NME6 (middle panels) or RCC1L (right panels), and IP fractions were probed for OPA1, RCC1L and NME6. Both endogenous and NME6-FLAG isoforms co-immunoprecipitated RCC1L, and inversely RCC1L co-immunoprecipitated endogenous NME6 and the NME6-FLAG isoforms, thus demonstrating the presence of stable NME6/RCC1L complexes (Fig. 10). There was no co-IP of OPA1 under any condition, thus excluding stable NME6/OPA1 complexes. Interestingly, FLAG-tagged NME6 did not pull-down endogenous NME6 (Fig. 10a, b), thus confirming the absence of physical interaction between NME6 monomers, consequently impairing the formation of higher oligomers.

## Discussion

The numerous members within the NME/NDPK protein family evidently raise the question of their specific function(s) within a cell ([56] and related review series [57, 58]). A first issue is their cellular distribution, since they partially localize to different cellular compartments (e.g. cytosol, nucleus, mitochondria) and, even more importantly, interact with specific proteins, lipids or DNA. In this way, those NMEs possessing NDPK activity can locally supply or fuel GTP to GTPases or G proteins. However, several other molecular mechanisms have been described for specific NMEs, including protein histidine phosphorylation, lipid transfer, or DNA damage repair. Few attentions have been given so far in respect to localization and function to Group II NMEs. In particular for NME6, information is largely limited to the two publications that initially reported NME6 in 1999 [35, 36], and which are partially contradictory. Mehus and coworkers predicted a protein of 186 amino acids (aa) [35], while Tsuiki and coworkers reported a 194 aa protein localized, at least partly, with mitochondria [36]. Further, Mehus et al. describe ubiquitous expression in body organs [35], while Tsuiki et al. find expression mainly in heart and skeletal muscle, placenta and pancreas, and detected NDPK activity [36]. Our study finally clarifies these issues by providing evidence for the existence of both NME6 isoforms (albeit at very different levels), for its ubiquitous expression, for a lack of NDPK activity, and for NME6 localization inside mitochondria. Importantly, we provide further detailed insight into NME6, showing that it is monomeric and lacks any phosphotransfer activity due to the absence of the active site histidine phosphorylation. We further localize NME6 mainly in the matrix, partially bound to MIM, and show that its overexpression reduces ADP-stimulated respiration and complex III abundance. We finally reveal its association network, and identify a stable interaction with RCC1L, a potential regulator of mitoribosome assembly and mitochondrial translation [44, 51].

By mass spectrometry, we could demonstrate the existence of both, the shorter 186 aa and the longer 194 aa isoform of NME6, differing in the first eight N-terminal amino acids. However, only the shorter NME6-186 is readily detectable by immunoblotting and thus abundant. Generation of both isoforms by forced expression of NME6-194-FLAG suggests translation from the same mRNA via the existing two ATG initiation sites. However, we cannot exclude alternating splicing that produces two different mRNAs, or post-translational processing of the nascent NME6-194 protein. NME6 was expressed in all cell lines of different origin that we examined. This supports a rather ubiquitous expression of NME6 as reported by Mehus and coworkers [35]. They detected appreciable amounts of NME6 mRNA in all 16 tissues examined, especially in kidney, prostate, ovary, intestine and spleen [35]. Ubiquitous NME6 expression is also supported by studies in Zebrafish [3, 33]. Here, NME6 was the most expressed gene among Group II NME members in ovary, highly expressed in particular during oogenesis and early development. Only Tsuiki and coworkers reported NME6 mRNA mainly in heart, placenta, skeletal muscle and pancreas, with very low levels in other organs like brain, lung, liver and kidney [36]. In summary, ubiquitous expression of NME6, even if it may be increased in some specific tissues or physiological processes, suggests a basic role of NME6 in some fundamental cellular process. Indeed, biallelic knock-outs (KO) of NME6 are lethal in early mouse development as reported in the public database of the International Mouse Phenotype Consortium [59]. In MDA-MB-231T cells, we could only generate monoallelic NME6 KO clones, but were unable to generate biallelic KO cells by using CRISPR-Cas9 technology. This also suggests potential lethality of the latter condition.

A precondition of NDPK activity seems to be the presence of NME oligomers, either in the form of homo- or heterohexamers as in eukaryotes [45–47] or archaea and some bacteria such as *Bacillus subtilis* [60, 61], or in form of tetramers as in some other bacteria [62–64]. Using glutaraldehyde-crosslinking on recombinant NME6-His, we failed to detect more than traces of any multimeric species. Similar results were obtained on the sponge NME6Sd, where the hexameric form was negligible [39]. This is in striking contrast to the crosslinking of human NME1 or sponge NMEGp1Sd (ancestral Group I protein) performed by our group under the same conditions, resulting predominantly in hexamers, with other oligomers barely detectable [65]. Likewise, IP failed to provide evidence for any NME6 oligomers. Endogenous NME6 did not co-immunoprecipitate with either FLAG-NME6, or NMEs from Group I, namely NME1, NME2, NME3 and NME4, the latter two overexpressed as FLAG-tagged variants. The lack of interaction with NME1 and NME2 is expected since they both localize in the cytosol [66]. However, NME3 partially localizes to the outer face of MOM, while it is also found in cytosol and nucleus [26, 27], and NME4 mainly to the outer face of MIM with a smaller part in the matrix [30]. Although NME4 could co-localize with NME6, both proteins do not stably interact. In conclusion, NME6 does not form oligomers with itself or with Group I NME proteins. This lack of oligomerization could be a consequence of the additional stretch of > 20 amino acids at the NME6 C-terminal end as suggested by Munier and coworkers for NME5 [67]. Consistent with the absence of oligomers, recombinant NME6-His did not show NDPK activity in the coupled pyruvate kinase-lactate dehydrogenase assay, with NME1-His as positive control being fully active under these assay conditions. Interestingly, similar results were obtained for the sponge homologue NME6Sd, which also showed no activity compared to the NDPK active NMEGp1Sd [39, 65]. A critical intermediate of phosphotransfer is phosphorylation of the main histidine in the NDPKs active site that drives the ping-pong reaction mechanism. We used the recently developed anti-pHis antibodies [68, 69] to study histidine phosphorylation with endogenous NMEs. While a pHis signal was clearly co-migrating with NME1 in SDS-PAGE, this was not the case with NME6. These data are consistent with earlier studies that failed to detect NDPK activity or histidine phosphorylation in recombinant NME6, while pHis was present in NME1, NME2, NME4, NME5 and NME7 [68, 70]. Similar to our approach, Tsuiki and coworkers studied both autophosphorylation and NDPK activity by using ^32^P-ATP labeling and recombinant GST-NME6. They reported low but detectable autophosphorylation (30 times less than with NME1) and measurable transfer of ^32^P to CDP [36]. Most likely, these divergent data are due to the highly sensitive method and/or traces of contaminating bacterial NME. In conclusion, NME6 has a very low or null rate of histidine phosphorylation and, therefore, cannot produce measurable NDPK activity. Even more, the lack of a phosphohistidine precludes any phosphotransfer activity, including potential protein histidine kinase activity. The molecular basis of this is unclear, since the NME6 active site has all the amino acid residues essential for kinase activity. However, a critical obstacle could be the insertion of three amino acids (as compared to NME1) within the *Kpn* loop which borders the catalytic site. Indeed, a valine residue in this loop (V112) participates in stabilizing the base of the nucleotide substrate [23]. Whether NME6 is only deficient in histidine phosphorylation and phosphotransfer activity, or also in nucleotide binding to the active site per se, remains to be shown.

Localization of NME6 in mitochondria has been evident from immunocytochemistry in fixed or live cells ([36], our work), and also suggested by large-scale proteomics studies [28, 37, 38], but these studies did not distinguish the two NME6 isoforms. We used a GFP reporter system for each individual NME6 isoform in live cells, and observed localization and potential intracellular movements of either isoform by time-lapse microscopy. These data demonstrate that both isoforms stably localize to mitochondria. We independently confirmed, with cellular fractionation, the localization of endogenous NME6 in purified mitochondria depleted of contaminants. Moreover, by subfractionation of mitochondria, we identify NME6 as mainly associated with the MIM and the matrix, similar in distribution to PDH-E1 α, a matrix protein partially bound to MIM, or ATP synthase α, part of the peripheral F_1_ subcomplex of ATP synthase that faces the matrix side. In contrast, we did not find soluble NME6 in the IMS. Such distribution suggests that the largest part if not all of NME6 is localized in the matrix space, partially bound to the MIM. This is consistent with the detection of NME6 in a large-scale study of the matrix proteome [38], and its absence in the proteome of IMS [71] and in the proteome of MOM facing the cytosol [27].

To narrow down possible functions of NME6, we first studied key functions of mitochondria in oxidative ATP generation. Since NME6 KO cells were not available, we used clones stably overexpressing NME6 to study NME6 dose effects on mitochondrial key functions. Basal respiratory activity in digitonin-permeabilized cells with both complex I- and II-linked substrates was largely unaffected (LEAK), but ADP-stimulated respiration (OXPHOS) was decreased in all NME6 overexpressing clones, leading to decreased OXPHOS/LEAK ratio. Since the potential across MIM was unchanged in these clones, this decrease suggests a reduced OXPHOS capacity, without specifically affecting ATP-ADP exchange across MIM or ATP synthase, which would rather lead to an increase in membrane potential [72]. Consistent with the limited OXPHOS respiration, we observed a strong decrease of OXPHOS complex III at the protein level, and a trend for downregulation of complexes I and IV, but not of complex V (ATP synthase). NME6 silencing did not significantly impact the vast majority of examined parameters, possibly linked to uncomplete silencing. Strikingly, the only significant outcome was an increase in OXPHOS respiration, the opposite of what has been observed in KI clones.

**Fig. 11:**
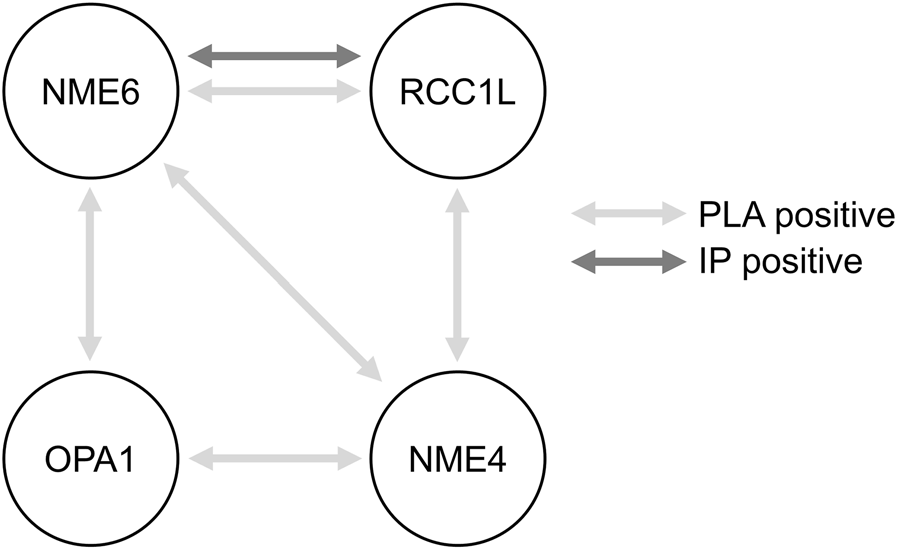
NME6 association and stable interaction with mitochondrial proteins. Summary of proximity ligation assay (PLA) and immunoprecipitation (IP) results obtained in our study for NME6 and three mitochondrial proteins: NME4, RCC1L and OPA1. A grey arrow indicates PLA positive association while a black arrow represents IP positive interaction

Our screen based on proximity ligation assays with a panel of mitochondrial candidate proteins gave further insight into potential NME6 functions. We identified OPA1, NME4 and RCC1L (WBSCR16) as being consistently close to NME6, thus associated in some manner (Fig. 11). Among those, we identified only RCC1L as a stable interactor, able to co-immunoprecipitate with NME6. Thus, RCC1L and NME6 are parts of the same complex, and indeed RCC1L is like NME6 localizing to the mitochondrial matrix, largely bound to MIM [38, 44, 51]. RCC1L is also the only NME6 interactor that has been detected consistently and with high score in different large-scale protein–protein interaction mapping studies [37, 51–54]. These used different methods, such as yeast two-hybrid or affinity purification followed by MS analysis, with NME6 as a bait and/or a prey. RCC1L is a mitochondrial member of the regulator of chromosome condensation 1 (RCC1) superfamily, named after the RCC1 protein, a guanine nucleotide exchange factor (GEF) for the RAN GTPase in the nucleus (reviewed in [73]). It came into focus only very recently due to its emerging role in the mitochondrial translation machinery. Although molecular details are not yet entirely clear, RCC1L interacts with both subunits of the mitochondrial ribosome and seems essential for mitoribosome assembly at MIM [44]. Both, RCC1L overexpression and silencing, lead to defects in mitoribosome biogenesis thus affecting mitochondrial translation [44]. One possible function of RCC1L is related to the pseudouridylation module, responsible for post-transcriptional modification of mitochondrial 16S rRNA, a component of the small ribosomal subunit [51, 74]. Our data also indicate a link of NME6 to mitochondrial translation, since overexpression of NME6 reduced the abundance of respiratory complexes to variable degrees, except for complex II, the only respiratory complex lacking mtDNA-encoded subunits. Interestingly, RCC1L was also localized in a second mitochondrial compartment, the IMS. Here, it was proposed as an interactor and a GEF of OPA1, a dynamin-related GTPase involved in MIM dynamics and mitochondrial fusion [55]. We found in earlier studies that mitochondrial NME4 interacts with OPA1 to locally fuel it with GTP [20, 31]. In the present study, we did not see stable interaction between NME6 and OPA1. We could neither confirm a stable interaction between RCC1L and OPA1 as reported earlier [55]. However, we detected association between NME6, OPA1, NME4, and RCC1L in PLA, suggesting that some NME6 may be present at the outer face of MIM, and that regulatory associations may exist between these proteins, without forming stable complexes. Still, the role of such regulations beyond those already known for the OPA1/NME4 complex remains unclear [75].

Collectively, our results and most other available data suggest that NME6 acts via its stable interaction with RCC1L in the mitochondrial matrix. A fundamental role of both proteins in eukaryotic cells is supported by the loss of NME6 [59] or the homozygous mutation of RCC1L [55], that both cause early embryonic lethality in mice. At the cellular level, RCC1L KO in HeLa cells increases apoptosis [55], and we were unsuccessful in generating homozygous NME6 KO in MDA-MB-231T cells. Further, both NME6 and RCC1L seem to be phylogenetically ancient genes [4, 73], even though the evolutionary history of RCC1L is not yet entirely clear. We thus propose a role of NME6 together with RCC1L, affecting mitochondrial translation that is required for respiratory complexes and ATP synthase. Indeed, RCC1L was initially identified by a genome-wide screen for genes essential for oxidative phosphorylation [51]. Consistent with this idea, NME6 overexpression negatively affected ADP-stimulated respiration and complex III abundance, but not the abundance of the entirely nuclear-encoded complex II. However, the exact roles of NME6 and RCC1L in these processes are only emerging, and their analysis is beyond the scope of this study.

## Conclusions

In this in-depth study of the human NME6 protein, we pinpointed the inability of NME6 to form oligomers, as well as its lack of histidine-phosphorylation, both supporting the lack of NDPK activity observed. We confirmed the mitochondrial localization of NME6 depicted previously, and further refined it as an inner membrane/matrix facing protein. NME6 overexpression affected mitochondrial respiration and abundance of respiratory complexes, suggesting a role in oxidative phosphorylation. Investigating NME6 proteins partners revealed a stable interaction with RCC1L (WBSCR16), a mitochondrial matrix facing protein involved in mitochondrial ribosome assembly and translation. Our research suggests a yet undescribed new role for NME6 protein in mitochondria, independent of the phosphotransfer activity. This work lays a solid foundation for future investigations on NME6 detailed molecular mechanisms and cellular impacts.

## Methods

### Plasmids

The cDNA sequences of the NME6 short and long isoform (186 aa and 194 aa, respectively) were extracted and cloned from Origene plasmids products RG200541 and RC200541, into pcDNA3.1, pET28b and pEGFP-N1 plasmids, using primers and restriction enzymes listed in the supplementary table (Additional File 1: Table. S1). Resulting proteins are tagged either with FLAG (DYKDDDDK), His (HHHHHH), or enhanced green fluorescent protein (EGFP) tags to comply with the design of individual experiments. The full-length NME4 cDNA sequence (187 aa) was extracted and cloned from pET28a( +)-NME4-FL [29] into pcDNA3.1 using primers and restriction enzymes listed in the supplementary table (Additional File 1: Table. S1). The full-length NME3 cDNA sequence (169 aa) was extracted and cloned from pCMVTag3-NME3-tetra-cys-tag (a kind donation of Prof. Thomas Wieland, Heidelberg University, Germany) into pcDNA3.1 using primers and restriction enzymes listed in the supplementary table (Additional File 1: Table. S1). The obtained pcDNA3.1-NME4-FL-FLAG and pcDNA3.1-NME3-FLAG plasmids were used for immunoprecipitation experiments. The pCFPmito plasmid was used in immunofluorescence to label mitochondria.

### Cell lines

Melanoma cell lines Mel 224, Mel 501, Mel 505, A375, A375M, WM793B, WM983B, LM6, CHL1, RPMI7951, were a kind donation of Dr. Bergamaschi (Barts and The London School of Medicine and Dentistry, London, UK) while MDA-MB-435 was purchased from ATCC^®^ CCL-2™. The sarcoma cell lines HTB 82 (rhabdomyosarcoma), HTB92 (liposarcoma), HTB93 (synovial sarcoma) and WT (2fTGH cells, variant of HT 1080 fibrosarcoma) as well as H1299 (lung carcinoma) were a kind donation of Dr. Neda Slade (Laboratory for Protein Dynamics, Ruđer Bošković Institute, Zagreb, Croatia). Detroit 562 (pleural effusion of pharyngeal carcinoma) and Cal 165 (spinocellular pharyngeal carcinoma), were a kind donation of Dr. Jeannine Gioanni, (Centre Antoine Lacasagne, Nice, France). MDA-MB-231T (pleural effusion of breast adenocarcinoma) was donated by Dr. Patricia S. Steeg (Center for Cancer Research, National Cancer Institute, USA; [40]). H460 (lung carcinoma) was a kind donation of Dr. Marijeta Kralj (Laboratory of Experimental Therapy, Ruđer Bošković Institute, Zagreb, Croatia). Other cell lines MDA-MB-436 (pleural effusion of breast adenocarcinoma), C33 (cervix carcinoma), LNCaP (prostate adenocarcinoma metastatic), PC3 (prostate adenocarcinoma metastatic), Du145 (prostate carcinoma, metastatic), MCF7 (pleural effusion of breast adenocarcinoma), SKOV 3 (ovary adenocarcinoma), RD (rhabdomyosarcoma), HCT 116 (colorectal carcinoma), SW620 (colorectal carcinoma, lymph node metastasis) and HeLa (cervix adenocarcinoma) were purchased from ATCC® CCL-2™. Non-cancerous cell lines HEK293 (human embryonic kidney), HACAT (Human keratinocytes), WPMY-1 (human prostate fibroblast) were purchased from ATCC^®^ CCL-2™ while MJ90 (HCA2) (human skin fibroblasts) cells were isolated previously from neonatal foreskin in the Pereira-Smith laboratory. With exception of MDA-MB-231T clones, cells were grown in DMEM (52100-021, Gibco) or RPMI (RPMI-XA, Capricorn Scientific) supplemented with 10% fetal bovine serum (FBS), 1% streptomycin-penicillin, 1 mM sodium pyruvate and 2 mM l-glutamine in a humidified chamber at 37 °C and 5% CO_2_. The MDA-MB-231T KI clones (KI-194-2, KI-186-1, KI-186-7, KI-186-18 and KI-186-26) were maintained in DMEM supplemented with 10% FBS, 1 mM sodium pyruvate, 2 mM L-glutamine and 1 mM G418. Cell lines were tested mycoplasma-free.

### Antibodies

For immunoprecipitation, NME6 (HPA017909 Sigma-Aldrich), NME1 (OP48 Calbiochem), RCC1L (SAB1401860 Sigma-Aldrich), irrelevant IgG-M (556648, BD Biosciences) and irrelevant IgG-R (2729P, Cell Signaling Technology) were used in combination with Dynabeads protein G (10003D, Invitrogen). For phospho-Histidine (pHis) immunoblotting, pHis antibodies (Rabbit hybridomas 1-pHis-SC1-1 and 3-pHis-SC44-1, kind donation of Dr. Tony Hunter, Salk Institute, La Jolla, CA, USA), NME6 (HPA017909 Sigma-Aldrich), and NME1 (UM800025 Origene) were used with HRP-anti-rabbit (7074 Cell Signaling Technology) and HRP-anti-mouse (7076 Cell Signaling Technology) secondary, all diluted in specific BSA blocking buffer (5% BSA (w/v) in TBST, pH 8.5). For other Western blots, antibody cocktail (Cytochrome *c*, GAPDH, PDH-E1 α, ATP synthase α, ab110415 Abcam), antibody cocktail OXPHOS (against complexes C-I to C-V, ab110412 Abcam), AK2 (AP8134B Abgent), β-actin (60008-1-Ig Proteintech), Calreticulin (C41720 BD Biosciences), FLAG (F1804 Sigma-Aldrich), Histone H3 (ab1791 Abcam; or #14269 Cell Signaling Technology), Lamp1 (15665 Cell Signaling Technology), NME1/2 (Nm23A&B kindly provided by Dr. I. Lascu and Dr. S. Volarević), NME6 (HPA017909 Sigma Aldrich), OPA1 (612607 BD Biosciences), RCC1L (SAB1401860 Sigma-Aldrich), RCC1L (ab247142 Abcam), Na^+^K^+^ ATPase (05-369 Merck Millipore), β-tubulin (2128S Cell Signaling Technology) and VDAC (a kind donation of Dr Marco Colombini) primary antibodies were used with HRP-anti-rabbit (7074 Cell Signaling Technology) and HRP-anti-mouse (7076 Cell Signaling Technology) secondary, all diluted in MILK blocking buffer (5% non-fat milk (w/v) in TBST). For PLA assays and immunofluorescence: NME6 (HPA017909 Sigma-Aldrich), NME4 (Milon et al. [29]), FLAG-tag (8146S Cell Signaling Technology), ANT (ab110322 Abcam), Drp1 (8570S Cell Signaling Technology), Mfn1/2 (ab56889/ab57602 Abcam), OPA1 (612607 BD Biosciences) and RCC1L (SAB1401860 Sigma-Aldrich) were used with IgG-Cy5 anti-mouse (ab2338714 Jackson), Dylight 488 anti-rabbit (ab96899 Jackson) and Alexa-Fluor488-anti-rabbit (A11008 Invitrogen) secondary antibodies.

### Recombinant protein expression, purification and thrombin cleavage

Recombinant proteins tagged with six histidine residues at the N-terminus were produced in *E.* *coli* strain BL21. Cells transformed with pET28b-NME6-186-His and pET28b-NME6-194-His were grown at 37 °C in LB medium until OD_600_ of 0.8, induced with 0.1 mM IPTG and grown overnight at 16 °C. Cells were washed and incubated on ice for 15 min in lysis buffer (50 mM HEPES, pH 7.4, 400 mM NaCl, 5 mM MgCl_2_, 10% glycerol (v/v), 10 mM imidazole, 1 mg/ml lysozyme). After sonication (8 × 30 s, 4 °C) and centrifugation (12,000 g, 1 h, 10 °C), the supernatants were applied onto metal affinity column (635502, Takara). After the washing step (washing buffer: 5 mM MgCl_2_, 50 mM HEPES pH 7.4, 400 mM NaCl, 10% glycerol, 10 mM imidazole), histidine tagged proteins were eluted (elution buffer: 50 mM HEPES pH 7.4, 300 mM NaCl and 150 mM imidazole). Amicon filters 10 kDa cutoff (UFC901024, Merck) were used to concentrate samples in storage buffer (25 mM HEPES, pH 7.4, 300 mM NaCl and 5 mM DTT). His-tag was removed from the NME6-194-His and NME6-186-His proteins using thrombin sepharose beads (7925-1, Biovision) according to manufacturers’ instruction. Briefly, 0.5 mg of recombinant proteins were incubated overnight at 4 °C with 7.5 µL of slurry in 50 mM Tris pH 8.0, 0.1 M NaCl. Protein concentration was assayed by Bradford method (500-0006, Bio-Rad). Samples were analyzed by Western blot.

### Recombinant protein crosslinking with glutaraldehyde

Recombinant protein NME6-186-His or NME6-194-His (9.5 µg) was mixed with 0.075% glutaraldehyde in crosslinking buffer (25 mM HEPES, pH 7.4, 300 mM NaCl, 5 mM DTT and 5 mM MgCl_2_) and incubated at 37 °C for 15 min. The reaction products as well as untreated proteins (control) were boiled 5 min at 95 °C and subjected to 12% SDS-PAGE, transferred to a PDVF membrane and visualized by protein staining with naphthol blue.

### Size-exclusion chromatography of recombinant proteins

Size-exclusion chromatography was performed at Biocentar d.o.o., Zagreb, Croatia on Akta avant 25 chromatography system (GE Healthcare). Recombinant proteins (NME6-186-His and NME6-194-His) were gel filtrated on Superdex 200 Increase 10/300 GL (GE Healthcare). The column was equilibrated with a mobile phase flow (10 mM NaH_2_PO_4_, 140 mM NaCl, pH 7.4) of one column volume (24 mL) followed by the injection of the sample. The sample was eluted with 1.5 volume of the column (36 mL). Fractions of 1 mL were taken at 2 min intervals. The chromatographic column was calibrated with the following Bio-Rad Gel filtration standards: thyroglobulin (670 kDa), γ-globulin (158 kDa), ovalbumin (44 kDa), myoglobin (17 kDa), and vitamin B12 (1.35 kDa).

### NDP kinase activity

NDPK activity was assayed on purified NME6-186-His and NME6-194-His recombinant proteins, and NME1-His [65] as control, using the standard pyruvate kinase-lactate dehydrogenase coupled assay, described by Agarwal and colleagues with minor modifications [2]. Experiments were performed at room temperature in 1 cm quartz cuvette containing 500 µL of reaction mixture composed of 50 mM Tris–HCl pH 7.5, 75 mM KCl, 5 mM MgCl_2_, 1 mg/mL bovine serum albumin, 1 mM phosphoenolpyruvate, 0.45 mM NADH, 1 mM ATP, 0.2 mM dTDP, 2 U of pyruvate kinase, 2.5 U of lactate dehydrogenase and 200 ng of recombinant NME protein. The reaction was started by addition of dTDP and absorbance was recorded every 10 s at 340 nm. The experiment was repeated six times (n = 6).

### Western blot

Proteins were extracted from mammalian cells in phosphate buffer saline (PBS) supplemented with protease inhibitors (11836170001 Roche). Pellets were sonicated (2 × 10 s, 4 °C) and protein concentration was determined by the BCA Protein Assay Kit (23227, Pierce). Proteins were boiled 5 min at 95 °C, separated on 10 or 12% SDS-PAGE (Tris–glycine based) and transferred to a nitrocellulose membrane. Membranes were stained either with ponceau red or naphthol blue. Primary antibody incubations were performed overnight at 4 °C, secondary antibody incubations were performed for 1 h at room temperature. Proteins were visualized with chemiluminescent reagent (NEL104001EA, Perkin-Elmer; 34096 and 34580, Thermo Scientific) using Alliance 4.7 imaging system (UVItec, Cambridge, UK). Phospho-His immunoblotting was performed according to the protocol published by Adam and coworkers, with minor modifications [23, 68]. All procedures were performed at 4 °C to preserve histidine phosphorylation. A fresh 12% SDS-PAGE (Tris–glycine based, stacking pH 7.4, resolving pH 8.8) was prepared. Gel, running buffer, transfer buffer, TBST and PBS were cooled down at 4 °C and adjusted to pH 8.2. The protein loading buffer (5 × LB = 10% SDS, 250 mM Tris–HCl, 0.02% bromophenol blue, 50% glycerol, 50 mM EDTA, 500 mM DTT) was cooled down, adjusted to pH 8.8 and diluted in PBS to 2 × LB on the day of experiment. MDA-MB-231T cells were grown until 90% confluency in a 10 cm dish, washed twice with PBS and scrapped in 500 µL of 2 × LB. The sample was incubated 10 min on ice before being sonicated (3 × 10 s, 4 °C) and clarified by centrifugation (14,000*g*, 10 min, 4 °C). The supernatant was carefully collected and equally divided in two parts, one incubated on ice to preserve histidine phosphorylation (4 °C), the other boiled (95 °C) for 10 min just before loading to lose histidine phosphorylation. After transfer, the nitrocellulose membrane was blocked (5% BSA (w/v) in TBST, pH 8.5), incubated with primary antibodies overnight at 4 °C and with secondary antibodies for 2 h at 4 °C. Proteins were visualized with chemiluminescent reagent (NEL104001EA, Perkin-Elmer; 34096 and 34580, Thermo scientific) using Alliance 4.7 imaging system (UVItec, Cambridge, UK).

### Fluorescence staining

For NME6 immunofluorescent staining associated to localization, HeLa cells were seeded in 8 well chambers (Ibidi, Gräfelfing) and transfected with pCFPmito using Lipofectamine 2000 (11668019, Invitrogen). Twenty-four hours post transfection the cells were rinsed with ice-cold PBS and fixed with 2% formaldehyde for 10 min at room temperature, followed by permeabilization with 0,1% Triton X-100. The cells were incubated with primary NME6 antibody overnight at 4 °C. The next day cells were washed with ice-cold PBS and incubated with Alexa-Fluor488-anti-rabbit secondary antibody (1 h, room temperature, obscurity). The cells were mounted in mounting medium (DAKO, Glostrup, Denmark). For immunofluorescent staining associated to PLA, MDA-MB-231T cells were fixed with 3.2% paraformaldehyde, permeabilized with 0.2% Triton X-100 in PBS, and blocked with PBS containing 3% BSA and 0.1% Tween20 (blocking buffer) before incubation (1.5 h at 37 °C) with one or two primary antibodies freshly diluted in blocking buffer. After washing with PBS, cells were incubated with secondary antibodies (1 h, room temperature, obscurity). Slides were mounted with antifading medium (Vectashield, Eurobio) before image acquisition. For live cell imaging and time-lapse imaging, MDA-MB-231T were seeded in a 4 chamber glass bottom dish and transfected with pCFPmito, pNME6-194-GFP or pEGFPN1-NME6-186-GFP using Turbofect (R0531, Thermo Scientific). Before acquisition the cells were washed with PBS and stained with 5 nM of MitoTracker™ Deep Red FM (M22426, Invitrogen) for 20 min in incubator (37 °C, 5% CO_2_). Cells were analyzed 48 h post transfection for live cell imaging. Cells were analyzed 24 h post transfection for time-lapse recording, for a 24 h period.

### Confocal imaging of live and fixed cells

For immunofluorescent staining and live-cell imaging related to localization, confocal microscopy was performed using Leica TCS SP8 X FLIM or a Leica TCS CSU SP8 confocal microscope equipped with an HC PL APO CS2 63 × /1.40 oil objective, 405-nm diode laser, argon-gas laser and a supercontinuum excitation laser (Leica Microsystems, Wetzlar, Germany). The stage-top environmental control system was used for live-cell imaging to maintain the temperature at 37 °C and Leibovitz’s L-15 medium (21083-027, Gibco) was used to support cell growth in the environment without CO_2_ equilibration. For time-lapse imaging z-stacks of 10 planes were taken every 15 min for a period of 24 h. For confocal imaging of live and fixed cells the excitation wavelengths and detection ranges were as follows: 488 nm and 500–550 nm for EGFP and Alexa488; 644 nm and 655–705 nm for MitoTracker™ Deep Red FM; 405 nm and 430–500 nm for DAPI and 458 nm and 470–520 nm for CFP. The hybrid (HyD) detectors were operated in the gated mode in order to suppress parasite reflection from the bottom glass surface of the cell-culture dish. Imaging was performed in a sequential scanning mode. For PLA assays and parallel (co-)immunofluorescence, excitation wavelengths and detection ranges were as follows: 488 nm and 500–545 nm for DyLight 488 and Syto 13; 522 nm and 600–659 nm for PLA red probes; 638 nm and 650–690 nm for Cy 5. Fluorescence emissions were precisely collected by dichroic filters and spectral detectors. The images were acquired through the entire cells by the mean of z-stacks with a z-step of 1 μm, a confocal pinhole of 1 (Airy units) for all channels, with at least 3 randomly chosen fields per condition.

### Proximity ligation assay (PLA)

In situ PLA was performed using a Duolink kit (Sigma-Aldrich, France). Cells grown on chamber microscopy slides were fixed with 3.2% paraformaldehyde, permeabilized with 0.2% Triton X-100 in PBS, blocked with a Duolink blocking agent and incubated with primary antibodies. PLA probes (secondary antibodies tagged with DNA oligonucleotides) were added, and hybridization, ligation, amplification, and detection (using Duolink Detection Reagents Red) were carried out according to the manufacturer’s protocol. Briefly, incubation with PLA probes was performed in a preheated humidified chamber for 1 h at 37 °C followed by ligation (30 min at 37 °C) and amplification (1 h 40 min at 37 °C). Nuclei were stained with 2.5 µM Syto13 (ThermoFisher, France) for 15 min, and image acquired by confocal microscopy.

### Stable clones overexpressing NME6-186-FLAG and NME6-194-FLAG

MDA-MB-231T cells were transfected with pcDNA3.1, pcDNA3.1-NME6-194-FLAG and pcDNA3.1-NME6-186-FLAG using Turbofect transfection reagent (R0531, Thermo Scientific) according to manufacturers’ instruction. The stable clone selection started 24 h post transfection in DMEM supplemented with G418 for 14 days. Selected cells were diluted and seeded at one cell per well in a 96-well plate. Single-cell colonies were selected under the microscope and allowed to grow in DMEM with G418. Viable colonies were saved and the plasmid’s random integration was confirmed by PCR for “empty” vector clones or by Western blot using anti-FLAG antibody for KI clones. Stable clones KI-186-7, KI-186-18 and KI-186–26 were used for analysis of mitochondrial mass, membrane potential and respiration, as well as PLA experiments. Stable clones KI-194-2 and KI-186-1 were used for evaluating NME6 protein expression in stable clones (Fig. 2b).

### Silencing of NME6 by siRNA

Silencing was performed using DharmaFECT 4 Transfection Reagent (T-2004-02, Dharmacon) and ON-TARGETplus Human NME6 siRNA—SMARTpool (L-006755-00-0005, Dharmacon) to silence NME6, or scramble siRNA (D-001810-01, Dharmacon) as a negative control, according to the manufacturers’ instruction. Briefly cells were grown to reach 30% confluency the day of transfection. Six hours post transfection the medium was changed and cells were allowed to grow until analysis, 72 h post transfection.

### Cell fractionation using a commercial kit

Cellular fractionation assay was performed using MDA-MB-231T cells and Cell Fractionation Kit (ab109719, Abcam), according to manufacturers’ protocol. Crude “Cytosolic”, “Mitochondrial” and “Nuclear” fractions were quantified using BCA Protein Assay Kit (23227, Pierce), and an equal amount of proteins from each fraction was analyzed by Western blot.

### Isolation and purification of mitochondria

MDA-MB-231T cells were grown to 80% confluency, and 350 × 10^6^ cells were used for the experiment. The whole procedure of mitochondrial isolation and purification was performed on ice as described earlier [30, 76] with minor modification. Cells were scrapped in PBS and centrifuged 5 min at 750 g. Collected cells were resuspended and combined in 10 mL of BufferA (210 mM mannitol, 70 mM sucrose, 0.2 mM EDTA, 10 mM HEPES pH 7.5). Cells were homogenized by 10 passages through a 25G needle. Homogenate (H) was centrifuged 5 min at 2,000 g. The supernatant (S1) was kept on ice and pellet, resuspended again in 10 mL BufferA, underwent 6 additional passages through a 25G needle and centrifugation (5 min, 2000 g). Pellet enriched in nuclei (P) was resuspended in 5 mL of BufferA and kept on ice until analysis. The supernatant was combined with S1 and centrifuged 10 min at 13,000 g. The resulting supernatant enriched in cytosol (C) was kept for analysis. The pellet was resuspended in 1.5 mL of BufferB (210 mM mannitol, 70 mM sucrose, 0.1 mM EGTA, 10 mM HEPES pH 7.5) and centrifuged 5 min at 500 g. The supernatant was transferred in a clean tube and centrifuged 10 min at 10,000 g. The resulting pellet enriched in crude-mitochondria (MC) was resuspended in 1 mL of BufferB and purified on 25% Percoll gradient by ultra-centrifugation (35 min, 100,000 g, Beckman 60Ti fixed angle). Two bands were collected from bottom to top (MP, percoll-pure mitochondria; CON, contaminants) and washed in 10 mL of BufferB (10 min, 7,000 g). Resulting pellets were resuspended in 0.5 mL BufferB. Protein concentration was measured using Bradford method (500-0006, Bio-Rad) and 2 µg from each fraction was analyzed by Western blot.

### Mitochondrial subfractionation

The whole procedure of mitochondrial subfractionation was performed on ice using MC obtained from MDA-MB-231T cells as described above, and swelling-shrinking procedure as described earlier [77] with minor modification. After washing in BufferB (10,000 g, 10 min), MC pellet was resuspended in 200 µL of swelling buffer (SW1, 10 mM KH_2_PO_4_ pH 7.4) and incubated 20 min on the rotator. Then 200 µL of shrinking buffer (SW2,10 mM KH_2_PO_4_ pH 7.4, 30% sucrose, 30% glycerol, 10 mm MgCl_2_, 4 mM ATP) was added and the suspension was incubated for further 1 h on the rotator. The sample was gently sonicated (2 × 15 s) in a water bath sonicator and centrifuged at 12,000 g for 10 min. The resulting low-speed supernatant (MCss_ls) was kept on ice while the mitoplast-containing low-speed pellet (MCss_lp) was washed two times in BufferA (10 min, 12,000 g) before being resuspended in 400 µL of BufferSW1 and sonicated using ultrasonic homogenizer with a metal tip (Bandelin, Germany; 3 × 15 s, 60 s cooling interval). Both MCss_ls and MCss_lp were centrifuged 1 h at 160,000 g (Beckman ultracentrifuge; 70.1Ti fixed angle rotor). Resulting high-speed pellets (respectively MCss_ls_hp and MCss_lp_hp) were resuspended in 100 µL BufferB, while resulting high-speed supernatants (respectively MCss_ls_hs and MCss_lp_hs) were separately concentrated using microconcentrator YM-3 (Millipore, 42,403) according to manufacturer’s instruction. Protein concentration was determined by Bradford method (500-0006, Bio-Rad), and 2 µg of protein were analyzed by Western blot.

### Multiple reaction monitoring mass spectrometry

The liquid chromatography-mass spectrometry (LC–MS) analysis was performed (Biocentar d.o.o., Zagreb, Croatia) on 1290 Infinity LC System (Agilent Technologies, USA) coupled with 6460 Triple Quad Mass Spectrometer (Agilent Technologies, USA). In silico digestion of NME6-194 and NME6-186 sequences by trypsin was performed using *Skyline* software (v. 3.7.0.10940.). The difference in isoforms was obtained within the following sequences: MTQNLGSEMASILR for the NME6-194 isoform and MASILR for the NME6-186 isoform. Total HeLa and MDA-MB-231T cell lysates were digested with trypsin (γ = 1 mg/mL) for 18 h at 37 °C and 600 rpm (Digital Shaking drybath, Thermo Scientific, USA). Acquity UPLC BEH separation column C18 1.7 µm, 2.1 × 150 mm (Waters, USA) was used for chromatographic peptide separation. Mobile phase A (0.1% (v/v) aqueous formic acid solution) and mobile phase B (acetonitrile) were both degassed in an ultrasonic bath. The separation was performed at 40 °C column temperature, 15 µL of sample was injected with a gradient flow of 0.3 mL/min starting at 95% A and decreasing to 60% A over 16 min. Mass spectra were recorded in a positive resolution mode with the capillary voltage set at 3.5 kV, at a gas temperature of 300 °C, and at a gas pressure of 40 psi. All measurements were performed in duplicate.

### Immunoprecipitation

MDA-MB-231T cells were transfected with pcDNA3.1-NME3-FLAG, pcDNA3.1-NME4-FLAG, pcDNA3.1-NME6-186-FLAG, pcDNA3.1-NME6-194-FLAG using Turbofect transfection reagent (R0531, Thermo Scientific). Cells were collected 48 h post transfection, lysed by sonication (2 × 10 s, 4 °C) in TEEN buffer (50 mM Tris pH 7.4, 0.5% NP40, 150 mM NaCl, 5 mM EDTA) supplemented with protease inhibitor (11836170001, Roche) and clarified by centrifugation (16,000 g, 20 min, 4 °C). The supernatant was recovered and used as input (I). Pull-down using FLAG-agarose (A2220, Sigma) was performed according to the manufacturers’ instruction. Briefly, 40 µL of slurry was washed and incubated overnight with 200 µg of input protein (I), on the rotator at 4 °C. The next day, supernatant (Sn) was saved while FLAG-agarose-Ag complexes were washed and eluted (IP). Immunoprecipitation using Dynabeads™ Protein G (10003D, Invitrogen) was performed according to the manufacturers’ instruction. Briefly, 50 µL of beads were washed and incubated 40 min with 2 µg of antibody, on the rotator at room temperature. NME1, NME6 and RCC1L antibodies were used to immunoprecipitate endogenous proteins while irrelevant IgG-M and IgG-R were used as negative controls. Beads-Ab complexes were washed and incubated with 200 µg of input protein (I) on the rotator, overnight at 4 °C. The next day, supernatant (Sn) was saved while beads-Ab-Ag complexes were washed and eluted (IP). In both immunoprecipitation experiments, the whole elution volume (IP) was loaded on SDS-PAGE, while 30 µg of input (I) and supernatant (Sn) were loaded and analyzed by Western blot.

### Mitochondrial membrane potential, mass, network characteristics and respiration

MDA-MB-231T cells (Ctrl, stable clones overexpressing NME6-186-FLAG) were grown until 90% confluence, detached with trypsin, and resuspended. Each suspension was distributed into two tubes and either incubated for 15 min with 50 nM Mitotracker GreenFM (Life Technologies, ThermoFisher Scientific, USA) or 50 nM TMRM (tetramethylrhodamine methyl ester, Life Technologies, ThermoFisher Scientific, USA) in a 5% CO_2_ humidified atmosphere at 37 °C and protected from light. Cell suspensions were immediately analyzed by FACS (BD LSR FORTESSA, Becton Dickinson, France) with excitation at 488 nm or 532 nm and emission band-pass filters 530/30 nm or 585/15 nm for Mitotracker GreenFM or TMRM, respectively. Mitochondrial mass was estimated by quantification of Mitotracker labeling. TMRM-labelled cells were further incubated for 15 min with 250 µM CCCP (carbonylcyanide m-chlorophenyl hydrazone, Sigma-Aldrich, France), and the mitochondrial membrane potential calculated as a difference of TMRM fluorescence before and after CCCP addition, normalized to mitochondrial mass. Confocal images of Mitotracker GreenFM-stained cells were used for quantification of mitochondrial shape and network parameters as described [43].

Oxygen consumption was measured in a thermostatically controlled Clark electrode oxygraph at 37 °C (Strathkelvin MS200A system). Detached cells were counted and resuspended at 100 × 10^6^ cells/mL on ice. An aliquot of 5 × 10^6^ cells was added in the oxygraph chamber containing KET buffer (150 mM KCl, 1 mM EGTA, 20 mM Tris pH 7, 2) and inorganic phosphate (5 mM) to give a final volume of 500 μl. Then digitonin (50 µg/mL) was added and incubated for two minutes to allow permeabilization of the plasma membrane. Oxygen consumption of cells was measured with glutamate (5 mM) and malate (2.5 mM), or with succinate (5 mM) as substrates (LEAK state [78]), as well as after addition of ADP (0,5 mM; OXPHOS state [78]) and after the addition of cytochrome *c* (10 μM; control for intact mitochondrial membranes). Results are expressed as nmol O_2_ consumed per minute and per 5 × 10^6^ cells.

## Supplementary Information

**Additional file 1: Table. S1** Plasmids used in the study.

**Additional file 2: Figure. S1** Stable clones express a high amount of exogenous NME6-186-FLAG. **a** Ten micrograms of cell lysate from MDA-MB-231T (Ctrl) and stable clones overexpressing NME6-186-FLAG (KI-186-7, KI-186-18, KI-186-26) were analyzed by Western blot, using NME6 antibody. **b** Quantifications of endogenous NME6 (Ctrl) or exogenous NME6-186-FLAG (KIs) by densitometry were normalized to naphthol blue. Band intensities are displayed as a ratio of the hatched Ctrl sample. Note: The exogenous NME6-186-FLAG expression is ten to twenty time higher than the endogenous NME6 in Ctrl. Endogenous NME6 is undetectable in stable clones.

**Additional file 3: Figure. S2** Mass spectrometry reveals the presence of two isoforms of the endogenous NME6 in HeLa and MDA-MB-231T cells. Mass spectrometry analysis of **a**, **b** HeLa and **c, d** MDA-MB-231T cell lysate was designed to detect separately **a, c** NME6-186 and **b, d** NME6-194 endogenous isoforms. Experiments were performed in duplicate (n = 2). Both NME6 long and short isoforms are detected in cells, without information about their relative abundance.

**Additional file 4: Movie. S1** Time-Lapse microscopy of HeLa cells overexpressing NME6-186-GFP. HeLa cells were transiently transfected with pEGFPN1-NME6-186-GFP (green) 24 h before acquisition. Cells were stained with MitoTracker™ to label mitochondria (red) 20 min before acquisition. Images were recorded every 15 min for a 24 h period. A long-term fluorescence overlay indicates stable localization of NME6 protein with mitochondria.

**Additional file 5: Movie. S2** Time-Lapse microscopy of MDA-MB-231T cells overexpressing NME6-186-GFP. MDA-MB-231T cells were transiently transfected with pEGFPN1-NME6-186-GFP (green) 24 h before acquisition. Cells were stained with MitoTracker™ to label mitochondria (red) 20 min before acquisition. Images were recorded every 15 min for a 24 h period. A long-term fluorescence overlay indicates stable localization of NME6 protein with mitochondria.

**Additional file 6: Movie. S3** Time-Lapse microscopy of RKO cells overexpressing NME6-186-GFP. RKO cells were transiently transfected with pEGFPN1-NME6-186-GFP (green) 24 h before acquisition. Cells were stained with MitoTracker™ to label mitochondria (red) 20 min before acquisition. Images were recorded every 15 min for a 24 h period. A long-term fluorescence overlay indicates stable localization of NME6 protein with mitochondria.

**Additional file 7: Figure. S3** Endogenous NME6 localizes in mitochondrial enriched fraction. MDA-MB-231T cells were fractionated by a commercial, detergent-based method yielding cytosol-enriched (C), mitochondria-enriched (M) and nuclei-enriched (N) fractions. Ten micrograms of protein were analyzed by Western blot using antibodies specific for different cellular compartments (Mitochondria: ATP synthase α, PDH-E1 α; Cytosol: GAPDH; Nucleus: Histone H3).

**Additional file 8: Movie. S4:** 3D projections of the Mitotracker Green-stained MDA-MB-231T control cell (Ctrl) shown in Fig. 7a.

**Additional file 9: Movie. S5:** 3D projections of the Mitotracker Green-stained MDA-MB-231T stable clone overexpressing NME6-186-FLAG protein (KI-186-7) shown in Fig. 7a.

**Additional file 10: Movie. S6:** 3D projections of the Mitotracker Green-stained MDA-MB-231T stable clone overexpressing NME6-186-FLAG protein (KI-186-18) shown in Fig. 7a.

**Additional file 11: Movie. S7:** 3D projections of the Mitotracker Green-stained MDA-MB-231T stable clone overexpressing NME6-186-FLAG protein (KI-186-26) shown in Fig. 7a.

**Additional file 12: Figure. S4:** Analysis of the mitochondrial network. Panel of confocal images of MDA-MB-231T cells stained with Mitotracker Green: **a** wild-type cells (Ctrl), cell clones stably overexpressing NME6-186-FLAG about 10-times (KI-186-7) or about 20-times (KI-186–18 and -26) as compared to endogenous NME6 in Ctrl, or **b** cells transfected with scramble siRNA (siCtrl) or with siRNA against NME6 (siNME6) (all scale bars: 10 µm). Peripheral regions of the mitochondrial network (shown as magnified images) were used for quantification of network parameters. The most relevant parameter, the elongation factor, is shown in **c** for KI clones and **d** for silenced cells. All data are given as mean ± SEM (n = 8).

**Additional file 13: Figure. S5:** NME6 knock-down slightly increases ADP-stimulated respiration. **a** Immunoblot of MDA-MB-231T cells transfected with scramble siRNA (siCtrl) or transfected with siRNA against NME6 (siNME6) using NME6 antibody. **b** Densitometry analysis related to (a). Bands intensities normalized to ponceau red signals are shown as a ratio of the hatched siCtrl sample. Note: NME6 band intensity in silenced cells represent roughly 15% of NME6 band intensity in the control cells. **c** Confocal images of MDA-MB-231T cells stained with Mitotracker Green, either cells transfected with scramble siRNA (siCtrl) or cells transfected with siRNA against NME6 (siNME6) (scale bar: 10 µm). **d-g** Oxygraphy analysis of respiration in the cells shown in (c) that were digitonin-permeabilized and supplied with substrate (LEAK, grey) and stimulated with ADP (OXPHOS, black). **d** Cellular oxygen consumption with glutamate/malate. **e** OXPHOS/LEAK ratios for (d). **f** Cellular oxygen consumption with succinate. **g** OXPHOS/LEAK ratios for (f). **h** Mitochondrial mass determined by Mitotracker Green staining. **i** Mitochondrial membrane potential determined by TMRM and corrected for mitochondrial mass (for details see Material and Methods). All data are given as mean ± SEM (n > 10 for (d-g), n = 3 for (h-i)). For comparison between siCtrl and siNME6 cells, significance is given as ** p < 0.01; * p < 0.05 (Student’s test).

**Additional file 14: Figure. S6** Relevant controls for proximity ligation assays. Proximity ligation assays (PLA) and immunofluorescence were performed on untransfected MDA-MB-231T cells (Ctrl) and a clone stably expressing NME6-186-FLAG (KI-186-18). **a** PLA negative controls using a single antibody only, defining the PLA-negative background. **b** Immunofluorescence staining showing reactivity of antibodies at concentrations used for PLA assays. Note: OPA1 is expressed at only low levels in MDA-MB-231T cells (unpublished data). (All scale bars: 10 µm).

## Data Availability

The datasets used and/or analyzed during the current study are available from the corresponding author on reasonable request.

## Abbreviations

AK2: Adenylate kinase 2
ANT: ADP/ATP translocase
CFP: Cyan fluorescent protein
CON: Contaminants
Ctrl: Control
DRP1: Dynamin-related protein 1
EGFP: Enhanced green fluorescent protein
GAPDH: Glyceraldehyde-3-phosphate dehydrogenase
GEF: Guanine nucleotide exchange factor
GST: Glutathione S transferase
HRP: Horseradish peroxidase
IMS: Intermembrane space
IP: Immunoprecipitation
KI: Knock-in
KO: Knock-out
KPN: Killer of prune
LC–MS: Liquid chromatography mass spectrometry
MC: Crude mitochondria
MFN: Mitofusin
MIM: Mitochondrial inner membrane
MOM: Mitochondrial outer membrane
MP: Percoll-pure mitochondria
MS: Mass spectrometry
NDPK: Nucleoside diphosphate kinase
OPA1: Optic atrophy 1
PDH-E1 α: Pyruvate dehydrogenase E1 component subunit alpha
pHis: Phospho-histidine
PLA: Proximity ligation assay
RCC1: Regulator of chromosome condensation 1
RCC1L: RCC1-like G exchanging factor-like protein
VDAC: Voltage-dependent anion-selective channel
WBSCR16: Williams-Beuren syndrome chromosomal region 16 protein
