# Supplementary material for: NME6 is a phosphotransfer-inactive, monomeric NME/NDPK family member and functions in complexes at the interface of mitochondrial inner membrane and matrix
Source: Cell Biosci. 2021 Nov 17;11:195. doi: 10.1186/s13578-021-00707-0 (PMC8597243; doi:10.1186/s13578-021-00707-0)
Supplement: Supplementary file 1 — Additional file 1: Table. S1 Plasmids used in the study. [file 13578_2021_707_MOESM1_ESM.docx]

| **Plasmid Name** | **Plasmid of origin** | **Cloned in** | **Primers/restriction site** | **TAG** | **Host expression** |
| --- | --- | --- | --- | --- | --- |
| pNME6-194-GFP | RG200541, Origene | / | / | Turbo-GFP, C-ter | Mammalian |
| pEGFPN1-NME6-186-GFP | RC200541, Origene | pEGFPN1 | XhoI 5´-GTCTAGCTCGAGTAATGGCCTCAATCTTGCG-3´  EcoRI 5´- CTAGACGAATTCGGCTGGTCCTAGGCC -3´ | eGFP, C-ter | Mammalian |
| pET28b-NME6-194-His | RC200541, Origene | pET28b | NdeI 5’-GTCTAGCATATGACCCAGAATC-3’  BamHI 5’-CTAGACGGATCCTCAGGCTGGTCCTAGGCC-3’ | 6xHis, N-ter, in front of Thrombin cutting site | Bacterial |
| pET28b-NME6-186-His | RC200541, Origene | pET28b | NdeI 5’-GTCTAGCATATGGCCTCAATC-3’  BamHI 5’-CTAGACGGATCCTCAGGCTGGTCCTAGGCC-3’ | 6xHis, N-ter, in front of Thrombin cutting site | Bacterial |
| pcDNA3.1-NME6-194-FLAG | RC200541, Origene | pcDNA3.1 | BamHI 5´-GTCTAGGGATCCACGAGATGACCCAGAATCTGGGG-3´  EcoRI 5’-CTAGACGAATTCTTAAACCTTATCGTCGTCATCCTTGTAATCGGCTGGTCCTAGGCC -3´ | FLAG, C-ter | Mammalian |
| pcDNA3.1-NME6-186-FLAG | RC200541, Origene | pcDNA3.1 | BamHI 5´- GTCTAGGGATCCACGAGATGGCCTCAATCTTGCG -3´  EcoRI 5´-CTAGACGAATTCTTAAACCTTATCGTCGTCATCCTTGTAATCGGCTGGTCCTAGGCC -3´ | FLAG, C-ter | Mammalian |
| pcDNA3.1-NME4-FL-FLAG | pET28a(+)-Nme4-FL  (Millon et al., 2000) | pcDNA3.1 | BamHI 5’-GTCTAGGGATCCACGAGATGGGCGGCCTCTTCTGGCGCTCC-3’  EcoRI 5’-CTAGACGAATTCTTACTTATCGTCGTCGTCCTTGTAGTCGGCTGGGTGGATGCTGCTGTG-3’ | FLAG, C-ter | Mammalian |
| pcDNA3.1-NME3-FLAG | pCMVTag3-Nme3-tetra-cys-tag  (Kind donation of Prof. Thomas Wieland) | pcDNA3.1 | BamHI 5’-GTCTAGGGATCCACGAGATGGACTACAAGGACGACGACGATAAGATGATCTGCCTGGTGCTGACC-3’  EcoRI 5’-CTAGACGAATTCTTACTCATACAGCCAGTGCC-3’ | FLAG, N-ter | Mammalian |
| pCFPmito | Kind donation of Dr. Yaron Shav‐Tal, Bar‐Ilan University, Ramat‐Gan, Israel) | / | / | CFP, directed to mitochondria | Mammalian |

**Tab. S1** Plasmids used in the study
