# Supplementary figures and images for: NME6 is a phosphotransfer-inactive, monomeric NME/NDPK family member and functions in complexes at the interface of mitochondrial inner membrane and matrix
Source: Cell Biosci. 2021 Nov 17;11:195. doi: 10.1186/s13578-021-00707-0 (PMC8597243; doi:10.1186/s13578-021-00707-0)

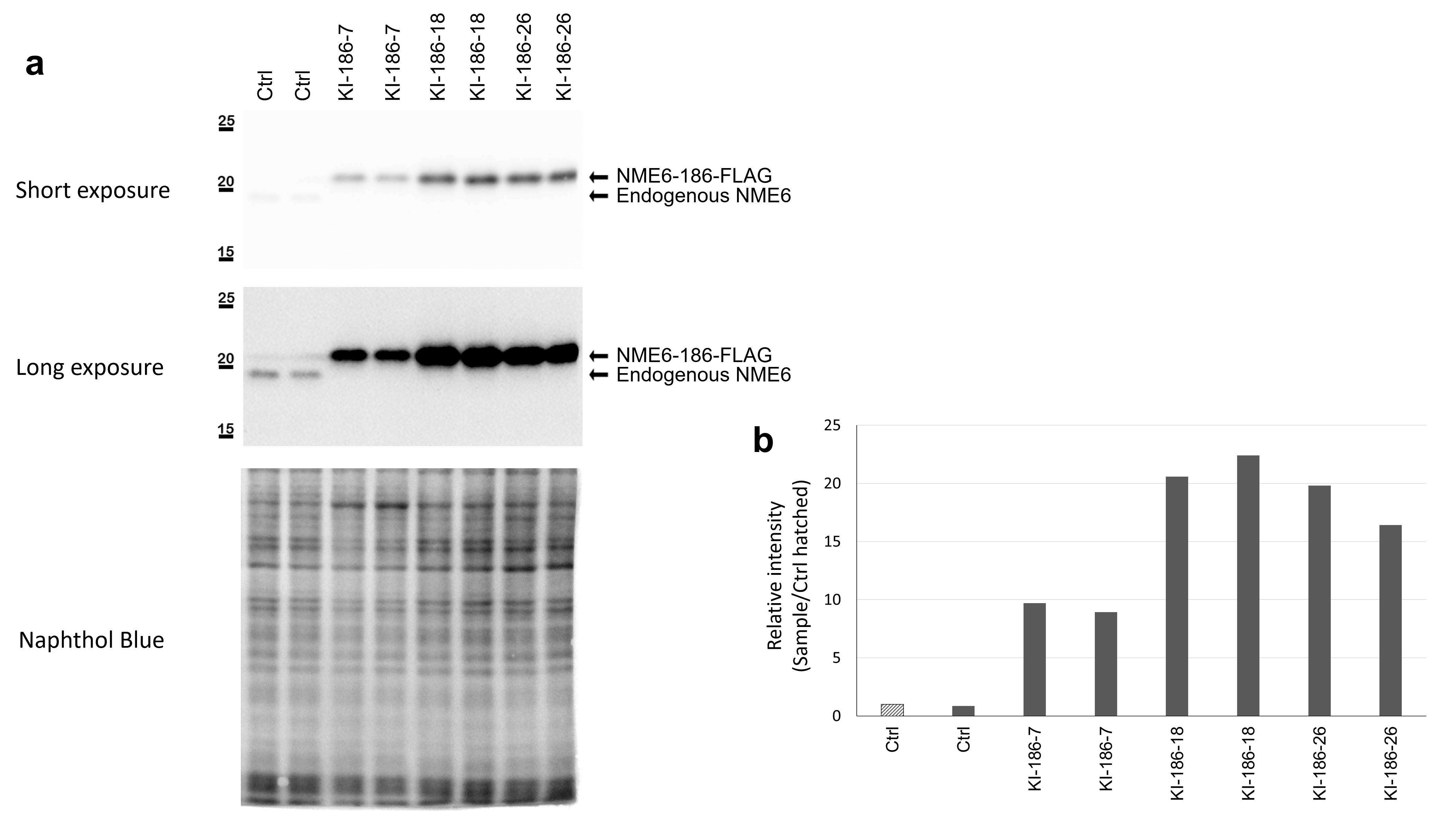

Supplement: Supplementary file 2 — Additional file 2: Figure. S1 Stable clones express a high amount of exogenous NME6-186-FLAG. a Ten micrograms of cell lysate from MDA-MB-231T (Ctrl) and stable clones overexpressing NME6-186-FLAG (KI-186-7, KI-186-18, KI-186-26) were analyzed by Western blot, using NME6 antibody. b Quantifications of endogenous NME6 (Ctrl) or exogenous NME6-186-FLAG (KIs) by densitometry were normalized to naphthol blue. Band intensities are displayed as a ratio of the hatched Ctrl sample. Note: The exogenous NME6-186-FLAG expression is ten to twenty time higher than the endogenous NME6 in Ctrl. Endogenous NME6 is undetectable in stable clones. [file 13578_2021_707_MOESM2_ESM.tif]

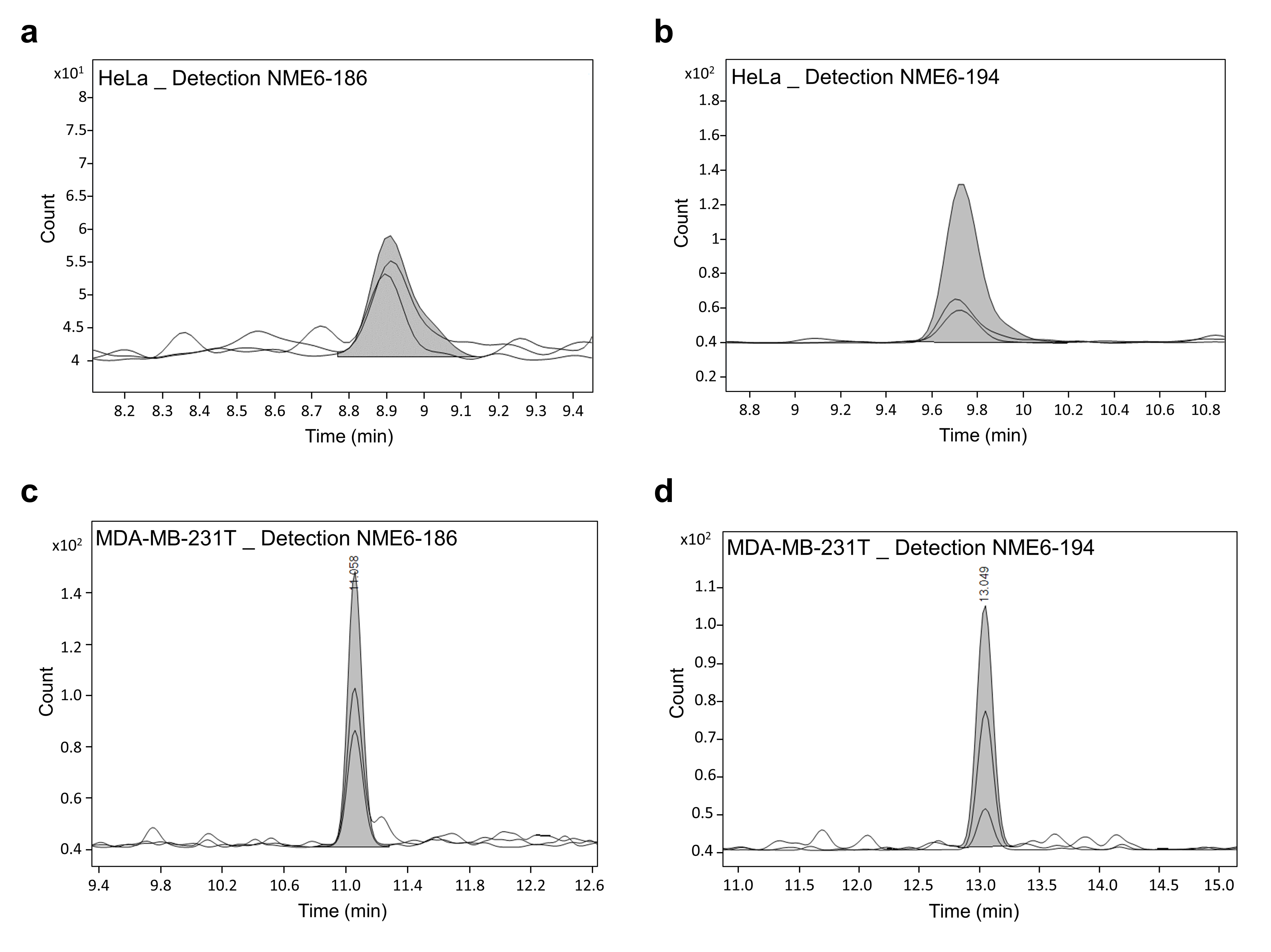

Supplement: Supplementary file 3 — Additional file 3: Figure. S2 Mass spectrometry reveals the presence of two isoforms of the endogenous NME6 in HeLa and MDA-MB-231T cells. Mass spectrometry analysis of a, b HeLa and c, d MDA-MB-231T cell lysate was designed to detect separately a, c NME6-186 and b, d NME6-194 endogenous isoforms. Experiments were performed in duplicate (n = 2). Both NME6 long and short isoforms are detected in cells, without information about their relative abundance. [file 13578_2021_707_MOESM3_ESM.tif]

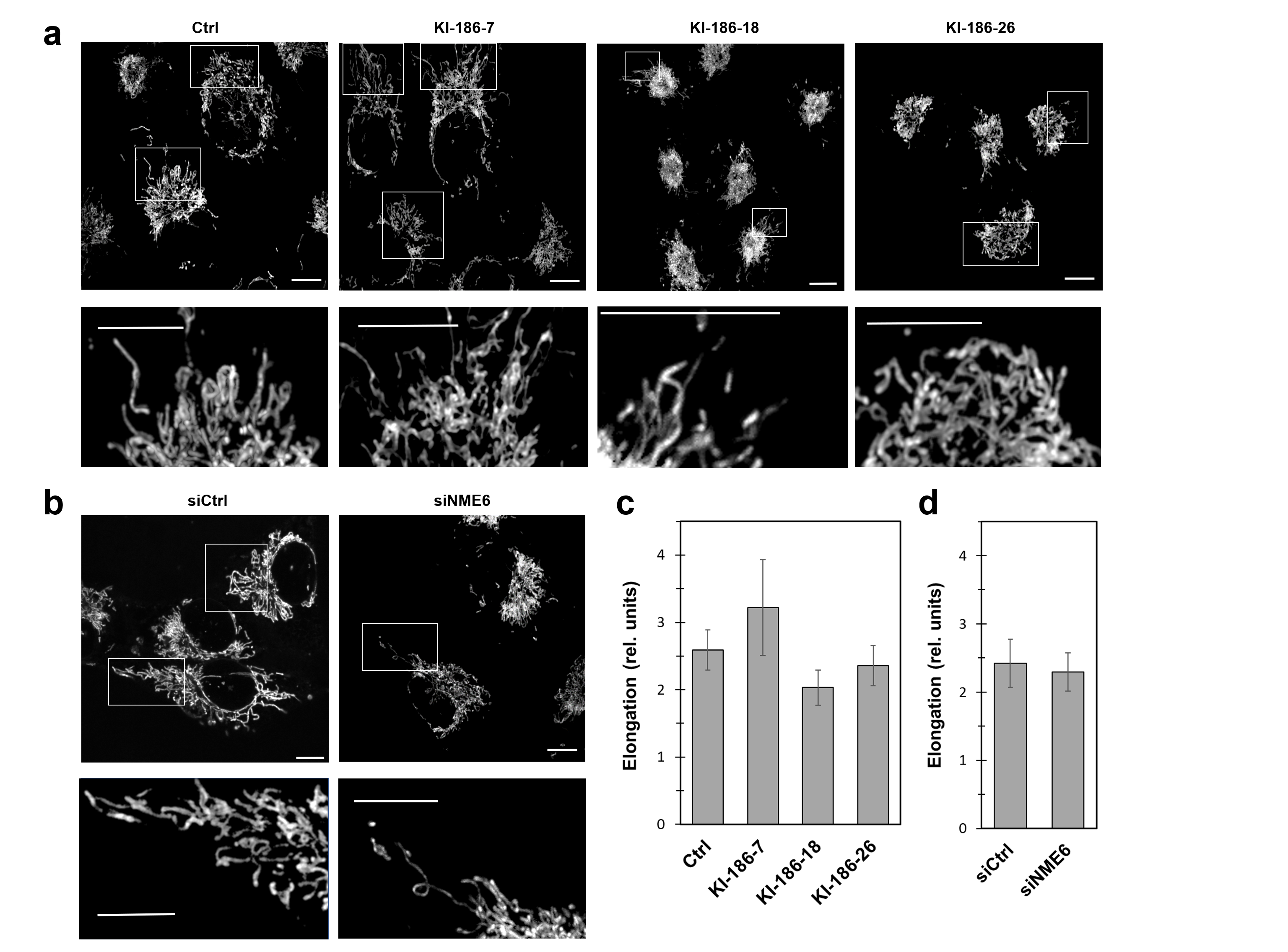

Supplement: Supplementary file 12 — Additional file 12: Figure. S4: Analysis of the mitochondrial network. Panel of confocal images of MDA-MB-231T cells stained with Mitotracker Green: a wild-type cells (Ctrl), cell clones stably overexpressing NME6-186-FLAG about 10-times (KI-186-7) or about 20-times (KI-186–18 and -26) as compared to endogenous NME6 in Ctrl, or b cells transfected with scramble siRNA (siCtrl) or with siRNA against NME6 (siNME6) (all scale bars: 10 µm). Peripheral regions of the mitochondrial network (shown as magnified images) were used for quantification of network parameters. The most relevant parameter, the elongation factor, is shown in c for KI clones and d for silenced cells. All data are given as mean ± SEM (n = 8). [file 13578_2021_707_MOESM12_ESM.tif]

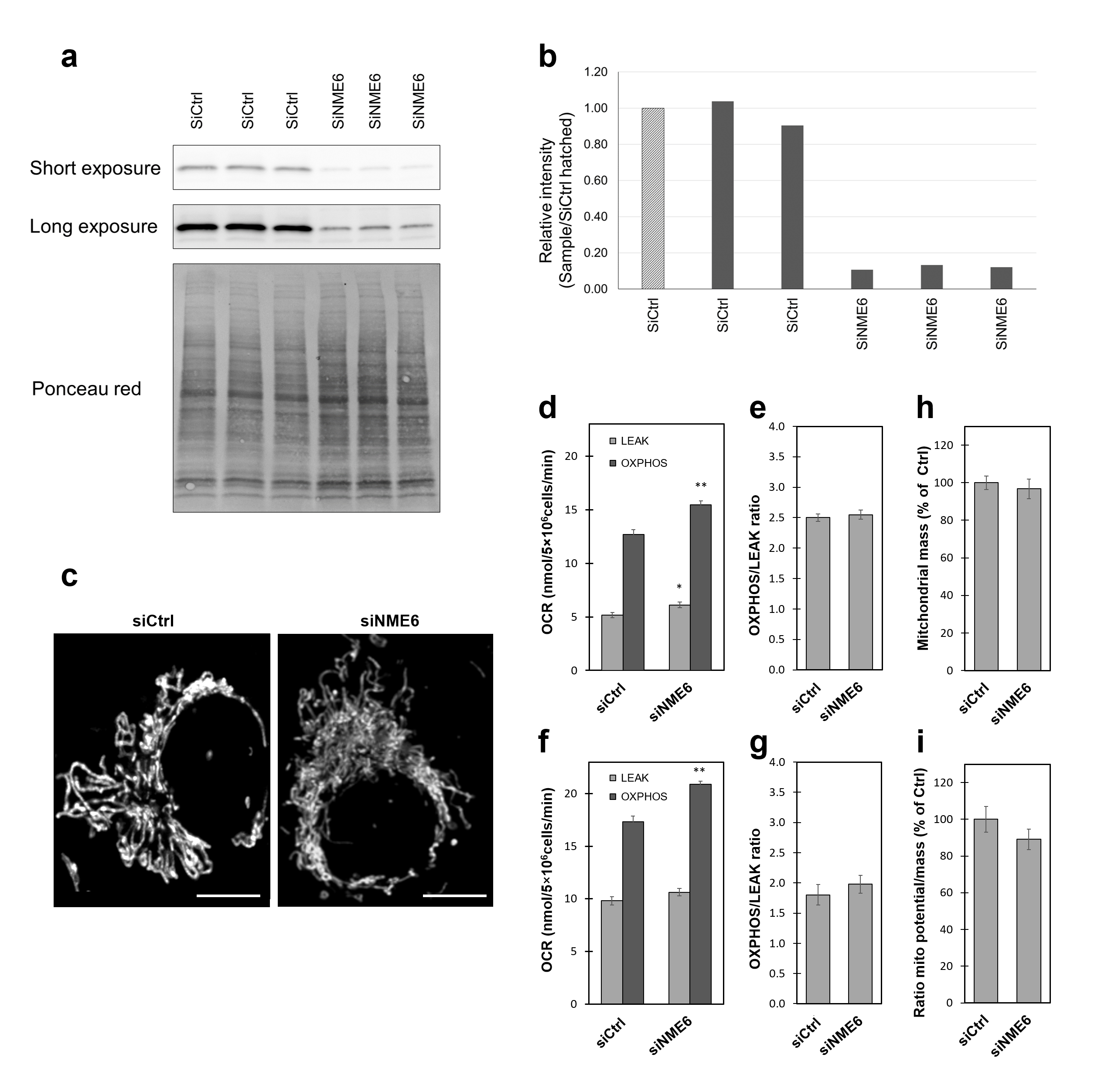

Supplement: Supplementary file 13 — Additional file 13: Figure. S5: NME6 knock-down slightly increases ADP-stimulated respiration. a Immunoblot of MDA-MB-231T cells transfected with scramble siRNA (siCtrl) or transfected with siRNA against NME6 (siNME6) using NME6 antibody. b Densitometry analysis related to (a). Bands intensities normalized to ponceau red signals are shown as a ratio of the hatched siCtrl sample. Note: NME6 band intensity in silenced cells represent roughly 15% of NME6 band intensity in the control cells. c Confocal images of MDA-MB-231T cells stained with Mitotracker Green, either cells transfected with scramble siRNA (siCtrl) or cells transfected with siRNA against NME6 (siNME6) (scale bar: 10 µm). d-g Oxygraphy analysis of respiration in the cells shown in (c) that were digitonin-permeabilized and supplied with substrate (LEAK, grey) and stimulated with ADP (OXPHOS, black). d Cellular oxygen consumption with glutamate/malate. e OXPHOS/LEAK ratios for (d). f Cellular oxygen consumption with succinate. g OXPHOS/LEAK ratios for (f). h Mitochondrial mass determined by Mitotracker Green staining. i Mitochondrial membrane potential determined by TMRM and corrected for mitochondrial mass (for details see Material and Methods). All data are given as mean ± SEM (n > 10 for (d-g), n = 3 for (h-i)). For comparison between siCtrl and siNME6 cells, significance is given as ** p < 0.01; * p < 0.05 (Student’s test). [file 13578_2021_707_MOESM13_ESM.tif]

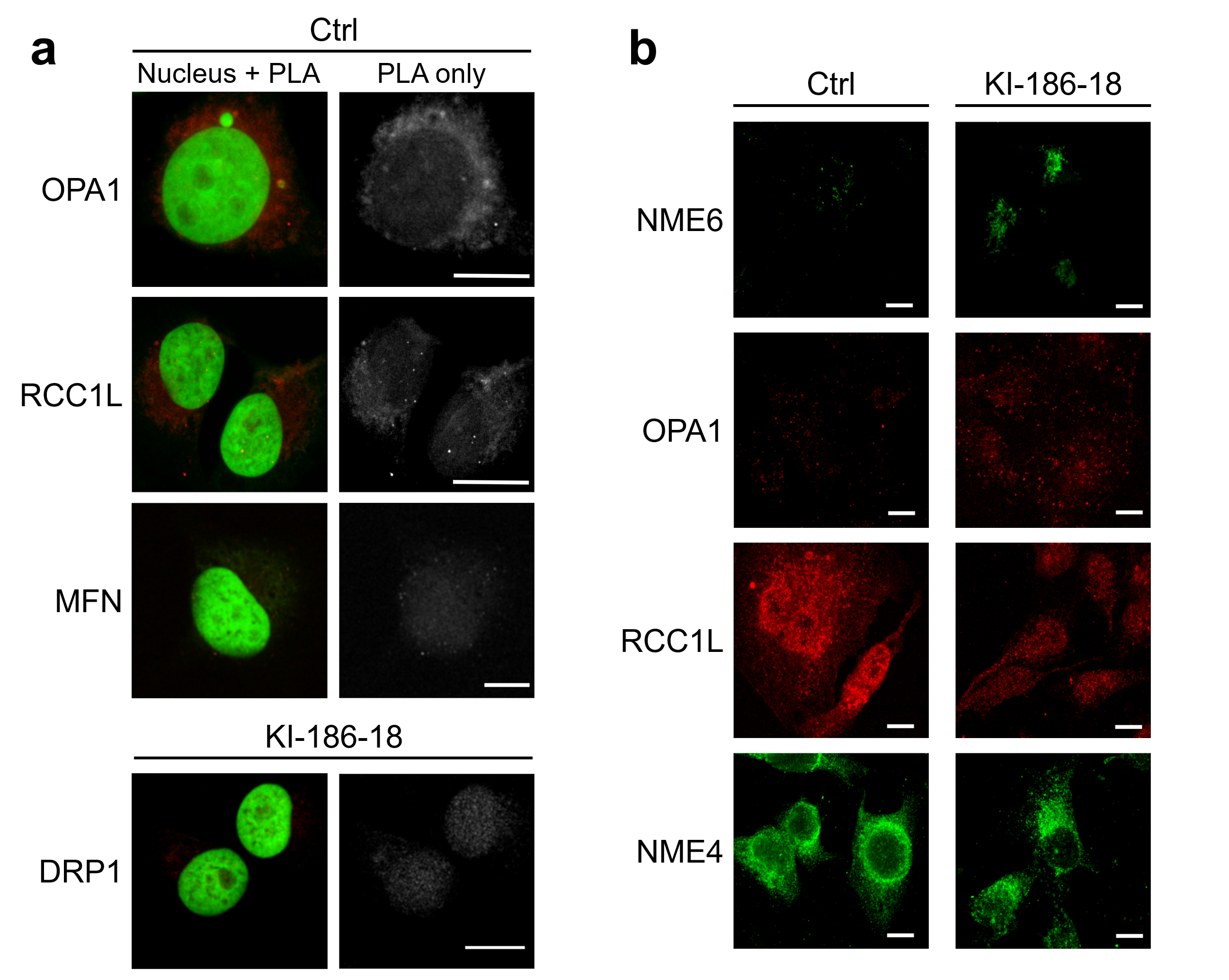

Supplement: Supplementary file 14 — Additional file 14: Figure. S6 Relevant controls for proximity ligation assays. Proximity ligation assays (PLA) and immunofluorescence were performed on untransfected MDA-MB-231T cells (Ctrl) and a clone stably expressing NME6-186-FLAG (KI-186-18). a PLA negative controls using a single antibody only, defining the PLA-negative background. b Immunofluorescence staining showing reactivity of antibodies at concentrations used for PLA assays. Note: OPA1 is expressed at only low levels in MDA-MB-231T cells (unpublished data). (All scale bars: 10 µm). [file 13578_2021_707_MOESM14_ESM.tif]
